# Advancement in the Breeding, Biotechnological and Genomic Tools towards Development of Durable Genetic Resistance against the Rice Blast Disease

**DOI:** 10.3390/plants11182386

**Published:** 2022-09-13

**Authors:** Parmeshwar K. Sahu, Richa Sao, Devendra K. Choudhary, Antra Thada, Vinay Kumar, Suvendu Mondal, Bikram K. Das, Ljupcho Jankuloski, Deepak Sharma

**Affiliations:** 1 Department of Genetics and Plant Breeding, Indira Gandhi Krishi Vishwavidyalaya, Raipur 492012, Chhattisgarh, India; 2 ICAR-Indian Agriculture Research Institute, Pusa, New Delhi 110012, India; 3 ICAR-National Institute of Biotic Stress Management, Baronda, Raipur 493225, Chhattisgarh, India; 4 Nuclear Agriculture and Biotechnology Division, Bhabha Atomic Research Centre, Mumbai 400085, Maharashtra, India; 5 Plant Breeding and Genetics Section, Joint FAO/IAEA Centre, International Atomic Energy Agency, 1400 Vienna, Austria

**Keywords:** rice, blast disease, *Magnaporthe oryzae*, R-genes, conventional breeding, molecular breeding, genome editing, genomic tools, broad-spectrum resistance

## Abstract

Rice production needs to be sustained in the coming decades, as the changeable climatic conditions are becoming more conducive to disease outbreaks. The majority of rice diseases cause enormous economic damage and yield instability. Among them, rice blast caused by *Magnaportheoryzae* is a serious fungal disease and is considered one of the major threats to world rice production. This pathogen can infect the above-ground tissues of rice plants at any growth stage and causes complete crop failure under favorable conditions. Therefore, management of blast disease is essentially required to sustain global food production. When looking at the drawback of chemical management strategy, the development of durable, resistant varieties is one of the most sustainable, economic, and environment-friendly approaches to counter the outbreaks of rice blasts. Interestingly, several blast-resistant rice cultivars have been developed with the help of breeding and biotechnological methods. In addition, 146 R genes have been identified, and 37 among them have been molecularly characterized to date. Further, more than 500 loci have been identified for blast resistance which enhances the resources for developing blast resistance through marker-assisted selection (MAS), marker-assisted backcross breeding (MABB), and genome editing tools. Apart from these, a better understanding of rice blast pathogens, the infection process of the pathogen, and the genetics of the immune response of the host plant are very important for the effective management of the blast disease. Further, high throughput phenotyping and disease screening protocols have played significant roles in easy comprehension of the mechanism of disease spread. The present review critically emphasizes the pathogenesis, pathogenomics, screening techniques, traditional and molecular breeding approaches, and transgenic and genome editing tools to develop a broad spectrum and durable resistance against blast disease in rice. The updated and comprehensive information presented in this review would be definitely helpful for the researchers, breeders, and students in the planning and execution of a resistance breeding program in rice against this pathogen.

## 1. Introduction

Rice (*Oryza sativa* L.) is the primary staple food and main source of nutrition for 2.5 to 3.5 billion people in the World, especially those living in fast-growing low-income countries [1,2,3]. Moreover, it is the main source of income and employment for more than 200 million populations across the world [2,4]. Over time, rice production has been augmented significantly; though, it is insufficient to fulfill the demand of the increasing global population [5,6], which needs to be increased by 38% by 2030 with the limited arable lands and severity of biotic and abiotic factors [1,7,8]. It is well understood that rice production is severely constrained by various biotic factors (pests, weeds, diseases, etc.) and abiotic factors (drought, cold, acidity, heat, salinity, etc.) [7]. However, biotic factors, especially diseases, have a great impact on rice production, which restricts rice breeders from crossing the yield barriers. It has been estimated that among the various diseases in rice, fungal diseases can decrease the global annual rice production by 14% [1,9,10]. Factually, out of the various fungal diseases, rice blast caused by *Magnaporthe oryzae* is one of the most devastating and recurring diseases causing enormous yield losses up to 70% to 80% within a short span of time [11,12,13]. Based on the scientific and economic importance of this fungal pathogen, it has been placed in the top ten fungal pathogens in the world [9,14]. Interestingly, the fungus *Magnoporthe oryzae* obtains a wide spectrum in affecting the rice plant, right from the seedling to late vegetative stages involving leaves, nodes, collar, panicles, panicle neck, and roots. The pathogen produces eye-shaped lesions on leaves (leaf blast), leaf collars (collar blast), culms, culm nodes, panicle neck nodes (neck rot), and panicles (panicle blast), which vary in color and shape depending on varietal resistance, environmental conditions, and age of plants [12,14]. 

When looking at the destructive nature of the disease, utmost emphasis should be given to managing the infestation of blast disease with the help of various innovative and durable techniques and strategies such as the development of resistant cultivars having durable resistance, use of fungicides, optimum fertilizer doses and appropriate planting time, etc. Moreover, among these, breeding for disease-resistant rice varieties possessing a major resistance gene (R-gene) is the most efficient and sustainable approach to counter disease outbreaks due to its economic and environmental advantage [2,4,15]. Several strategies, viz., conventional breeding, mutation breeding, marker-assisted breeding, transgenic approaches, genome editing tools, etc., have been adopted so far for the development of disease-resistant cultivars [16], of which conventional breeding methods, including the pedigree method, backcross breeding, introduction and acclimatization, multiline breeding, pureline selection, recurrent selection, etc., are the robust and mostly used for developing novel genetic variants for blast resistance [17,18,19]. International Rice Research Institute (IRRI), Manila, Philipines, has developed many blast-resistant lines/donors for blast resistance through conventional breeding, which have made a significant impact on reducing the prevalence of blasts throughout the World [12,20]. Genetically diverse rice landraces with broad-spectrum resistance are valuable sources for the introgression of the resistance genes into rice cultivars for the control of blast, but the number is limited. Therefore, artificially induced mutants are an important resource for identifying new broad-spectrum resistant (R) genes/loci. In this aspect, physical/chemical mutagenesis is a way to develop new R genes/alleles, which can generate desirable resistant mutations that are free from the association of undesirable traits or linkage drag [21]. However, due to the quantitative nature of this resistance, conventional breeding takes a long time and requires many generations of crossing and screening to test the resistance, which can be overcome by adopting advanced molecular breeding methods and biotechnological tools [16]. 

With the advancement in plant genomics, breeders now have a wide spectrum of biotechnological tools to further strengthen the process of screening and developing blast-resistant rice varieties. Identification and mapping of a specific R gene for differential blast races in diverse elite germplasm/mapping populations with the help of DNA markers through association mapping/quantitative trail loci (QTL) mapping is a crucial step in ensuring the accuracy of their utilization in marker-assisted breeding (MAB) [15,16,22]. Until now, about 146 R-genes for rice blast resistance have been identified and mapped from both indica and japonica subspecies of rice, and about 36 genes have been molecularly characterized and cloned so far, which have boosted the breeding strategies for blast resistance in rice [15,23,24]. Marker-assisted gene pyramiding (MAGP) is one of the most appropriate approaches to confer horizontal resistance in the genotype for different races of blast pathogen by introgression of more than one resistance (R) gene [25,26]. Similarly, marker-assisted backcross breeding (MABB) is also an important method to transfer blast resistance in otherwise high-yielding rice varieties keeping all the characters of the same recipient parent. Marker-based selection methods are more accurate, reliable, and time-saving, do not require appropriate disease-favoring environmental conditions, and select the resistant genotypes even without inoculation of the pathogen [22,23,24]. Hence, these methods have been widely used by many plant breeders to develop blast-resistant varieties Worldwide [27]. 

Furthermore, genetic engineering techniques have enabled plant breeders to transfer blast-resistant genes from one organism to the background of other elite cultivars for developing resistant varieties [28,29,30,31]. However, the product of this method has not been widely accepted due to various safety and regulatory issues [29]. Recently genome editing technologies offer expanded potential for crop improvement as they allow specific alterations in DNA sequences that can be performed in vivo. These editing tools precisely manipulate specific sequences in the genome, which allows the insertion, deletion or substitution of nucleotides in specific genes or sequences [32,33]. Several genomic editing techniques, such as meganucleases (MNs), Zinc-finger nucleases (ZFNs), Transcription Activator–like Effector Nucleases (TALENs), and Clustered Regularly Interspaced Short Palindrome Repeats/CRISPR-associated protein 9 (CRISPR/Cas9), are used to promote specific genetic modifications. The development of the CRISPR/Cas9 technique has opened up a wide range of applications and could be explored in improving plant resistance to pathogens [34].

Breeding for disease resistance in rice is a critical component for increasing rice production and ensuring food security. Long-lasting and durable resistance for rice blast from a single gene is feasible but not often durable, as the pathogen can rapidly mutate and attack resistant cultivars. Broad host range, continuous genetic variation, evolution, and host shifts are the main reasons for the evolution of different types of races of Magnaporthe. It has made the development of resistant cultivars a daunting task. Therefore, the rice Magnaporthe interaction pathosystem has emerged as a model system to study host–pathogen interaction, genetics of rice blast resistance, host plant resistance, favorable conditions for disease occurrence, screening protocols, and conventional and modern breeding strategies to develop blast-resistant cultivars.

## 2. Economic Impacts of Rice Blast

Rice blast is one of the extensively dispersed plant diseases of a major food crop with huge destructive nature in Asian and African countries. It gradually becomes more troublesome to rice-growing farmers and threatens food security worldwide [35]. Therefore, significant efforts should be made to manage this disease to sustain the food security and economy of the World. The disease was first reported as “rice fever” in China by Soong Ying-shin in 1637, and later, it was reported in Japan by Imochi-byo in 1704 [36]. In India, it was first reported in Tamil Nadu in 1913 [37], which became more prevalent and devastating after the introduction of semidwarf and high-yielding varieties during the green revolution [38,39]. The first shattering epidemic was reported in 1919 in the Tanjore delta of south India [40]. 

Outbreaks of rice blast are the major persistent problem in more than 85 rice-growing countries of the World, especially in the South Asian and African countries [18], with annual yield losses ranging from 10 to 80% [1,11] depending upon the various factors such as varietal susceptibility, the degree of infection, the timing of fungicide application, high humidity, drought, heavy dew, high mean temperatures, high plant density and excessive nitrogen fertilizer [41]. Various studies stated that a total of USD 203.49 million is lost annually in terms of yield loss and management cost of rice blast disease, which alone could feed 60 million people around the World [1,15,42,43]. It has been estimated that the cost of management of blast diseases through chemical fungicides has exceeded USD 70/ha/year [43]. Interestingly, about 157 million tonnes of rice were lost globally between 1975 and 1990 by the severity of rice blast disease, which was more than 30% of global rice production. In India, when the disease accomplishes an epidemic form, yield losses due to rice blasts could be as high as 50%. During natural epidemics of blasts in the wet season, disease incidence ranged from 14 to 27% (above the economic threshold), resulting in yield loss of about 27–35 percent [23,42]. Severe epidemics of the blast occurred between 1980 and 1987 in Himachal Pradesh, Andhra Pradesh, Tamil Nadu, and Haryana, resulting in huge financial losses. According to an estimate, the extent of annual yield reduction caused by rice blast disease is adequate to feed around 60 million people each year [44]. In the United States of America, Arkansas, Louisiana, and Mississippi are the most affected states by the rice blast, where 6% to 50% yield loss has been recorded, and on average, a total of USD 69.34 million is lost annually due to blasts [43]. Moreover, rice blast is also a major issue in European countries such as Italy, Spain, Portugal, Greece, and France, where a 20% to 50% reduction in the milling yield has been observed [1,15,42,43].

## 3. Pathogenesis of the Causal Fungus *Magnoporthe oryzae*

**Figure 1:**
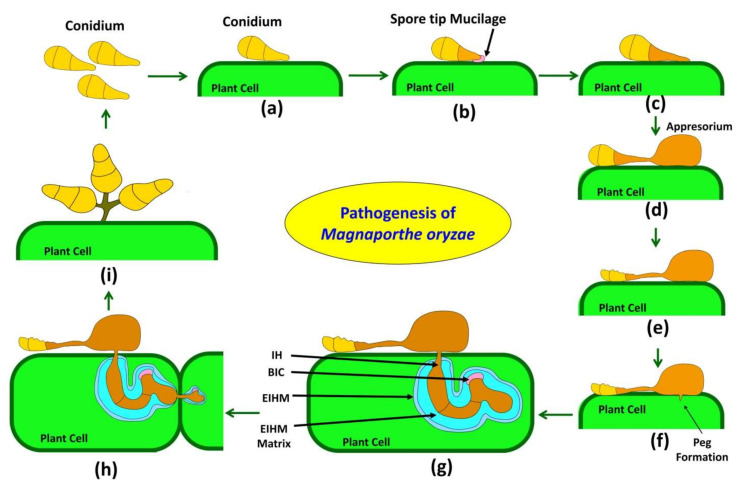
Pathogenesis of the rice blast fungus *Magnaporthe oryzae*. (**a**). Infection of the rice blast fungus starts when a three-celled conidium lands on the rice leaf surface. (**b**). Spore tip mucilage (STM) present on conidium helps the spore to attach to hydrophobic cuticle of rice plant. (**c**). Conidium germinate using food reserve and produce a narrow germ tube. (**d**). Germ tube elongates and give rise to appressorium. (**e**). Autophagy occurs in three-celled conidium and it dies in a programmed process. (**f**). In appressorium turgor pressure increases with the help of melanin layer on cell wall and synthesized glycerol inside. Then penetration peg forms at the base, punctured the cuticle of rice and allows entry into the epidermis of plant. (**g**). Plant tissue invasion occurs by means of bulbous, invasive hyphae (IH) that invaginate the rice plasma membrane and invade epidermal cells. Penetration peg develops into two primary hyphae and separated from rice cytoplasm by extra-invasive hyphal membrane (EIHM). Primary hyphae develop into invasive hyphae. At the tip of primary IH, a new structure known as biotrophic interfacial complex (BIC) develops which is present within EIHM. (**h**). Invasive hyphae moves from one cell to another by plasmodesmata. (**i**). Disease lesions occur on plant and sporulation starts under humid conditions, Spores develops on conidiophores with sympodially manners.

The blast disease of rice is caused by a filamentous fungal pathogen, *Magnaporthe oryzae* (previously *Magnaporthe grisae*). It is a hemibiotrophic fungus. In the early stage of pathogenesis, Magnaporthe behaves like a biotrophic pathogen and suppresses the plant immune system, and later it switches to a necrotrophic lifestyle that promotes necrosis of host cells [45]. Pathogenesis is a chain of events in a sequential manner that leads to the development of diseases such as attachment, pre-penetration activities, penetration, host recognition, infection, invasion, colonization, reproduction of pathogen, symptom development, dissemination, and survival of the pathogen [46]. Various steps involved in the process of pathogenesis are briefly described in subsequent paragraphs and also presented in Figure 1. 

### 3.1. Attachment and Germination 

*Magnaporthe oryzae* pathogenesis starts from a three-celled conidium (Figure 1a), which attaches itself tightly to a hydrophobic, waxy leaf cuticle of rice by an adhesive called spore tip mucilage (STM). STM exists in the periplasmic regions of the conidial tip cell before attachment (Figure 1b) [47]. Upon attachment, three celled conidia germinate and form unbranched germination tubes (also called germ tubes) from the apical cell and grow across the surface of the cuticle. Out of three cells of the spore, only basal and/or apical cells give rise to the germ tube; the middle cell seldom germinates and may function as a reservoir of energy for fungal growth on the host surface [48,49]. 

### 3.2. Germ Tube Elongation and Recognition of Host

Various chemical and physical cues, such as primary alcohols, cutin monomers, plant surface waxes, and hard and hydrophobic surfaces, trigger appressorium formation [50]. When the germ tube notices physical cues, such as hydrophobic and hard surface, the tip of the germ tube develops a dome-shaped appressorium which helps in pressure generation to penetrate the host surface. For surface recognition of host, pathogen implies several signal transduction pathways such as PMK1 mitogen-activated protein kinase (MAPK), cyclic adenosine monophosphate dependent protein kinase A (cAMP/PKA), and HOG1 signaling pathways, which also help in appressorium formation, infection peg formation, osmoregulation and cell wall integrity (Figure 1c) [51]. 

The pth11 gene encodes transmembrane protein Pth11, which is a G-protein coupled receptor (GPCRs). GPCRs are transmembrane receptors confined to the cell membrane of fungal spores and are involved in signal transduction from the outside environment to inside the cell, which helps pathogens to synchronize cell metabolism, cell transport, and growth [52]. Mutational disruption of pth11 triggered a failure in the maturation of appressorium, although the growth of germ tube hooks was not affected in the Δpth11 mutant and confirmed the role of Pth11 in pathogenesis [53]. GPCRs protein is composed of three G alpha (MagA, MagB, and MagC), one G beta (Mgb1), and one G gamma (Mgg1) subunits. Several mutational studies confirmed the role of subunits in pathogenesis [54]. Host cues and signals bind to the GPCR and activate downstream signaling cascade via G-protein, cAMP-PKA, and MAPK signaling pathways that influence metabolism, cellular growth, and morphogenesis of appressorium. A total of eight G protein signaling (RGS) proteins were identified (MoRgs1 through MoRgs8) in *Magnoporthe oryzae*. Surface hydrophobicity, conidiation, and mating are positively regulated by MoRgs1 and MoRgs4. For germ tube growth and appressorium formation, MoRgs1, MoRgs2, MoRgs3, MoRgs4, MoRgs6, and MoRgs7 are crucial. Although all RGS proteins are involved in the regulation of intracellular cAMP levels, only MoRgs1, MoRgs3, MoRgs4, and MoRgs7 are indispensable for complete virulence [55].

### 3.3. Appressorium Formation and Maturation 

Appressorium formation is regulated by a cascade of the gene in *Magnoporthe oryzae*. When a three-celled conidia land on the host surface, it attaches itself to the hydrophobic surface of the cuticle with the help of mucilage [56]. On germination, it forms a narrow germ tube in which a nucleus migrates and undergoes mitosis 4–6 h after germination. Various receptors present on the cell membrane of the germ tube recognize the host and initiate dome-shaped appressorium formation after the termination of the tip growth of the germ tube. Following two mitotic divisions, a daughter nucleus moves to develop appressorium, and the rest of the three nucleus returns to conidium and is degraded together with other spore contents, leaving a single nucleus in appressorium (Figure 1d) [57]. Turgor pressure generation through glycerol and other polyols synthesis inside appressorium and the formation of melanin layer between the cell membrane and the cell wall is known as appressorium maturation (Figure 1e) [45,58]. In *Magnoporthe oryzae*, cargo-independent autophagy and cell death were reported in three-celled conidium just before appressorium maturation to help turgor pressure generation in appressoria [59]. A total of sixteen genes were identified in non-selective macroautophagy; the mutation in any one gene leads to loss of pathogenicity in *Magnoporthe oryzae* [60]. Yin et al. [61] revealed the role of the autophagy-related gene (MoAtg1) of Magnaporthe, which encodes for kinase protein and possibly phosphorylates MoMkk1 to respond to endoplasmic reticulum stress during plant infection. Increased glycerol inside appressorium causes an influx of more water and builds tremendous pressure on the cell wall up to 8 MPa, and the melanin layer on the cell wall act as a barrier to the efflux of solute and helps to create structural rigidity to maintain increasing pressure [45]. Glycoprotein-rich mucilage adhesives glue melanized appressoria to host surfaces. Mucilage secreted around the base of the appressorium from the appressorial pore helps build up pressure on the host cuticle. Appressorium maturation and penetration are regulated by the MAPK signaling pathway mediated by an Mst11-Mst7-Pmk1 cascade [62]. The MAPK signaling cascade Mck1-Mkk1-Mps1 was reported to be involved in appressorium penetration, maintenance of cell wall integrity, and invasive growth of *Magnoporthe oryzae* [63]. 

### 3.4. Penetration Peg Formation and Invasion 

Turgor pressure generated by glycerol and melanized wall of appressorium is focused onto an unmelanized, thin-walled appressorium base where penetration peg starts to develop and generate tremendous pressure on host cuticle and rupture the rice cell cuticle (Figure 1f) [64]. A small amount of cell wall degrading enzymes (CWDEs) such as cutinase, Poly-galactorunase (PGs) is secreted by growing pathogen cells to weaken hard host surfaces [65]. The CUT2 gene of *Magnoporthe oryzae* produces a cutinase enzyme to degrade cutin present in the cuticle of rice [66]. Two homologs of PGs were identified in the genome of *Magnoporthe oryzae*, namely endo-PG (MGG_08938.6) and exo-PG (MGG_08752.6). Then, *Magnoporthe oryzae* obtains entry forcefully to the host cell by translating turgor force into physical force [67]. Ultra-structural analysis reveals that the appressorium pore of *Magnoporthe oryzae* is different from the rest of the appressorium, with the absence of melanin and a much thinner cell wall [68,69]. A turgor-sensing protein, Sln1, triggers downstream pathways when turgor pressure reaches a threshold, and the septin ring develops around the pore and acts as a barrier of lateral diffusion and control appressorium repolarization [70].

After penetration peg formation, rapid membrane biogenesis and F-actin polymerization occur at the penetration site [68,71]. Recent studies revealed the role of reactive oxygen species (ROS) burst in the re-modeling of cytoskeletal and rapid polymerization of F-actin. ROS burst is catalyzed by two genes, NOX1 and NOX2, that code for NADPH oxidase [71]. NOX1 gene plays a role in the maintenance of the polarized growth and organization of the toroidal F actin network and NOX2required for septin ring formation at the base of the appressorium during penetration peg formation [71,72]. Later appressorium nucleus migrates into the penetration peg, where it undergoes further rounds of mitosis; later, the penetration peg differentiates into primary infective hyphae, then globular invasive hyphae (IH) (Figure 1g). Fungal cells inside the host are confined by the plant-derived extra-invasive hyphal membrane (EIHM) to protect the host defense mechanism. This creates an enclosed apoplastic space between the pathogens IH and the cytoplasm of rice [72]. Further, the EIHM matrix contains several proteins, such as BAS4, which help plants generate defense mechanisms against the pathogen [72,73]. First invaded host cell filled by growth of IH before pathogen spreads into the neighboring host cell. IH switches back to primary IH and moves into uninfected neighboring cells through the plasmodesmata (Figure 1h) [72]. Later it again converted to IH in the newly infected adjacent cell. At the tip of primary IH, a new structure known as biotrophic interfacial complex (BIC) develops, which is present within EIHM. As the fungus multiplies within the first infected cell, BIC remains behind the bulbous IH and again reappears at the tip of the primary IH that will move into neighboring cells (Figure 1i) [73,74]. The mode of action of the focal BIC is unknown, but several effectors accumulate in the BIC to suppress the host immune responses and the virulence of *Magnoporthe oryzae* [72,74].

### 3.5. Invasion and Defense Suppression

*Magnoporthe oryzae* secretes a variety of effector proteins into the host cell to evade the immune response, manipulates host metabolism, and avoids recognition to take advantage during pathogenesis [74]. To date, many effector proteins identified in *Magnoporthe oryzae* interfere or interact with different target sites of rice. Most effector proteins are produced during the biotrophic phase [73]. Based on the secretion of the effector, there are two kinds of effectors in *Magnoporthe oryzae*. Apoplastic effectors are secreted into space between IH and EIHM, while Cytoplasmic effectors are secreted into BIC and then translocated into the host cytoplasm with the help of the exocyst complex and t-SNAREs. However, apoplastic effectors are secreted by the conserved ER (endoplasmic reticulum) to the Golgi secretory pathway [75]. Proved by treatment with Brefeldin A that interferes with Golgi-dependent secretion inhibited the secretion of apoplastic effectors such as Bas4 and Slp1 but did not affect the localization of cytoplasmic effectors Pwl2, Bas1, and Bas107 to the BIC [73]. 

Based on the recognition of effectors by a host protein, effectors are divided into two categories; the first category is Avr effectors, encoded by the avirulence (AVR) genes, which could be recognized by the corresponding resistance (R) gene of rice [76]. Another category is Non-AVR Effectors, which could not be recognized by the host R gene [76]. Effector proteins could be detected in up to four adjacent cells before the hyphal invasion to prepare the host cell for invasion [73]. There is a vast diversity in effectors’ structure; hence, the function of a few effectors is known to date. The biochemical function of Avr-Piz-t is known, which could suppress rice pathogen-associated molecular pattern (PAMP)-triggered immunity by obstructing the ubiquitin ligase activity of the rice RING E3 ubiquitin ligase APIP6 [77]. Well-characterized non-Avr effector Slp1 is a secreted LysM protein that accumulates in between IH and EIHM [73]. However, Slp1 is not required for appressorium penetration but is indispensable for hyphal growth in planta. Slp1 bind with the host component chitin elicitor binding protein (CEBiP) and defeat chitin-induced immune responses, as well as prevent the generation of ROS and defense-related gene expression in rice [73]. Over the past decades, developments in the functional identification of secreted effector proteins from *Magnoporthe oryzae* have remarkably enhanced our understanding of the molecular mechanisms involved in rice *Magnoporthe oryzae* interactions. More than 43 secreted proteins have been functionally identified in *Magnoporthe oryzae*, including 10 Avr effector proteins, PWL1, PWL2 [78], AvrPi-ta [79], AvrPiz-t [80], Avr-Pia, Avr-Pii, Avr-Pik/km/kp [81], Avr-CO39 [82], AvrPi9 [83], and AvrPib [84,85], four biotrophy-associated secreted proteins, BAS1 to BAS4 [86], five pathogenicity related secreted proteins, MPG1 [87], EMP1 [88], MHP1 [89], Slp1 [90] and MC69 [91]; 12 suppressors of plant cell death, IUG6, IUG9, NUP1, NUP2 and NUP3 [92], MoHEG13 [93], and SPD2, SPD4, SPD7, SPD8, SPD9 and SPD10 [94] and 20 plant cell death-inducing effectors proteins, MoHrip1 [95], MoCDIP1 to MoCDIP5 [96], MoHrip2 [96], MSP1 [97], MoNLP1, MoNLP2 and [98], MoSM1 [99] and MoCDIP6 to MoCDIP13 [100]. Recent efforts to understand fungal effector function have revealed that 50% of the *Magnoporthe oryzae* avirulence effectors and other fungal effectors belong to a new family of structurally conserved MAX effectors (Magnaporthe Avrs and ToxB) [101]. Interestingly, the vast majority of the *Magnoporthe oryzae* MAX effectors are expressed during the biotrophic stage of infection [101].

## 4. Pathogenomics of *Magnaporthe oryzae*

*Magnaporthe oryzae* is a hemibiotrophic fungus that is responsible for developing blast disease in rice. The genus Magnaporthe was created and accommodated in order Diaporthales [102]. It belongs to the phylum ascomycetes, which develop sexual spores known as ascospores for infecting host plants. *Magnoporthe oryzae* is known to have hundreds of pathotypes (races) that infect paddy. It is capable of causing damage in almost all stages of paddy, starting from the nursery stage up to grain formation [103]. In order to gain a better understanding of the genomics of this fungus, genome sequencing will play a major role in the future in developing varieties that will be resistant to multiple strains over different geographical regions [104]. Moreover, with the help of various advanced techniques such as genome editing and biotechnological innovations, any of the steps in the biological system of a pathogen can be disrupted or terminated to achieve resistance against blast disease [105]. Host-specific strains can be silenced using targeted mutations or RNAi techniques to create sustainable and strong resistance against the attack of *Magnoporthe oryzae*.

Interestingly, the genome of *Magnoporthe oryzae* has been sequenced firstly amongst various phytopathogenic fungi and is being utilized commonly as a model system to understand the mechanism of pathogenicity of pathogen and host–pathogen interactions. *Magnoporthe oryzae* showed huge genome instability due to the availability of recurrent repetitive sequences in its genome [42,106]. This genome instability plays an important role in the genome variation and the fast evolution of a new race of pathogens within the population. Until now, genome sequencing of more than 74 races of *Magnoporthe oryzae* has been completed. Among them, each strain contained isolated specific genes and genomic regions, which determine their racial evolution, environmental adaptation, chromosomal variability, variation in repeat element distribution, and host range specificity [96,107]. Consequently, *Magnoporthe oryzae* has a genome size of 40.12 Mb and contains 12,684 genes in the genome [42]. Further, one of the first strains to be completely sequenced was 70-15 using a whole-genome sequencing shotgun approach. It was discovered that this pathotype contained three MPAK pathways, which were associated with virulence [106]. Two strains of *Magnoporthe oryzae*, P131 and Y34, were sequenced using Sanger (2-fold) and 454 sequencing technologies [104]. Both of these races had approximately 13% unique DNA when compared with the previously sequenced laboratory strain 70-15. The interesting thing to note was that the deletion of a few genes would cause a decrease in the virulence capacity of the pathogen. For example, deletion of P131_scaffold00208-2 from P131 and Y34_scaffold00875-3 from Y34 would lead to a reduction in virulence and conidiation, respectively [104]. Paired-end libraries of FJ81278 and HN19311 strains of *Magnoporthe oryzae* were generated through Illumina sequencing. This helped in identifying many virulent genes which were different from 70-15, and genome variation was found at both the basic nucleotide level and chromosome level [96]. The whole genome assembly of another pathotype (RMg-Dl) was completed using PacBio Single-molecule and IlluminaHiSeq 2500 techniques. Strain RMg-Dl was isolated from the Swarna variety cultivated in the Bihar region of India [105].

## 5. Genetics of Blast Disease Resistance in Rice

**Figure 2:**
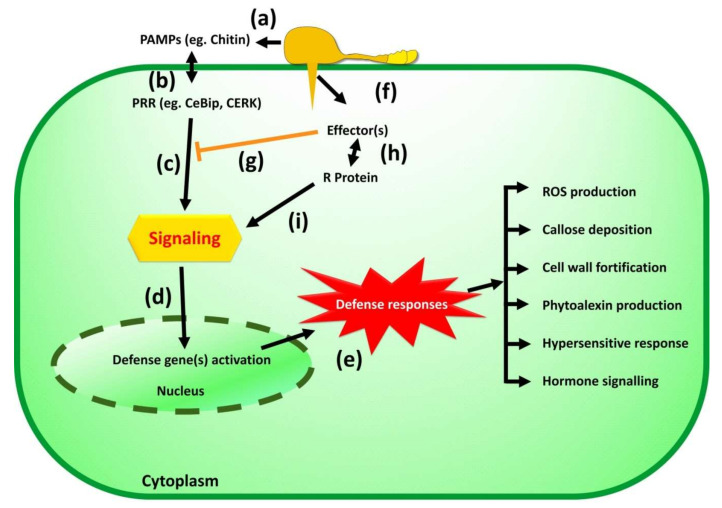
Overview of rice defense system against blast pathogen *Magnoporthe oryzae*. (**a**). PAMPs molecules present on membrane of Magnaporthe spore. (**b**). PRR presents on the rice cell membrane help to recognize PAMP molecules. (**c**). A successful recognition by PRR triggers PTI (PAMP-triggered immunity) and activates resistance signaling cascade. (**d**). Resistance signaling activates defense gene in nucleus of rice. (**e**). Defense responses includes ROS production, Callose deposition, Cell wall fortification, Phytoalexin production, Hypersensitive response, Hormone signaling etc. (**f**). In order to avoid recognition of PAMP molecules by PRR, Magnaporthe secretes effectors molecules. (**g**). Effectors molecules inhibit PTI responses which known as effector triggered susceptibility (ETS). (**h**). Plant resistant gene recognize effectors of pathogen which known as effector triggered immunity (ETI). (**i**). Successful recognition of effectors molecules by R gene activates resistance signaling cascade.

Understanding the genetics of the defense system generated by rice plants against the *Magnoporthe oryzae* is essential to designing a breeding program for developing the disease-resistant variety. A schematic representation of the ice defense system or immunity against blast pathogens is presented in Figure 2. Plants developed two layers of defense; in the first layer, pathogen-associated molecular patterns (PAMPs) are recognized by pattern recognition receptors (PRRs) present on an extracellular membrane or on a trans-membrane [108]. If PRRs of a plant can recognize PAMPs molecules of the pathogen, it induces a relatively weak basic immune response, known as PAMP triggered immunity (PTI), that obstructs the establishment of invading pathogen [109]. Further, the pathogen secretes effector protein to avoid or defeat triggered defense response, which is known as effector-triggered susceptibility (ETS) [110]. The second layer of plant defense is governed by resistance (R) proteins that recognize avirulence (Avr) effectors of the pathogen by direct or indirect binding and induce a wide array of defense responses; this response is known as effector-triggered immunity (ETI). A specific R protein binds to a specific AVR; hence, it is race-specific immunity [111]. 

PAMP molecule chitin present on the cell wall of *Magnoporthe oryzae* could be recognized by rice CEBiP (a chitin elicitor binding protein), a lysin motif (LysM) containing plasma membrane proteins, LYP4 and LYP6 [112]. In order to overcome this PTI response in rice, *Magnoporthe oryzae* secretes an effector protein, Secreted LysM Protein1 (Slp1), during the pathogenesis of new rice cells. Effector Slp1 accumulates in space between the rice plasma membrane and fungal cell wall and competes with CEBiP for binding of chitin molecules, and defeats plant defense gene expression and generation of reactive oxygen species [90].

R-proteins are multidomain proteins generally containing a nucleotide-binding (NB) and a leucine-rich repeat (LRR) domain. To date, 146 R genes, >500 QTLs against rice blast, have been identified, and 36 have been molecularly cloned and characterized [24,113,114,115]. Based on their structure, R genes can be categorized into different classes, i.e., CC-NBS-LRR, LRR, NBS NBS-LRR, Proline-containing protein, and Receptor kinase [116]. Very few pathogen AVR and rice R protein interactions are known, such as AVR1-CO39 to Pi-CO39, AVR-Pita to Pi-ta, ACE1 to Pi33, AVR-Pia to Pia, AVR-Pii to Pii, AVR-Pik/km/kp to Pik/Pik-m/Pik-p, AvrPiz-t to Piz-t, AVR-Pi9 to Pi9, AVRPib to Pib, and AVR-Pi54 to Pi54 [117].

The first studied interaction between AVR and R proteins in the *Magnoporthe oryzae*–rice pathosystem was AVR-Pita and Pi-ta. AVR-Pita is the first identified avirulence gene in *Magnoporthe oryzae*, encoding a predicted secreted protein that interacts with Pi-ta and triggers resistance [118]. Pi-ta codes a constitutively expressed 928 amino acidcytoplasmic NLR receptor which is an NBS-LRR class of R protein [119]. Effector AVR-Pita binds to the leucine-rich domain (LRD) of Pi-ta protein directly. A recent study reveals that in the absence of Pi-ta in rice, Avr-Pita targets the rice mitochondria and interacts with the OsCOX11 (Oryzae sativa cytochrome c-oxidase) assembly protein. OsCOX11 participates in mitochondrial reactive oxygen species (ROS) metabolism in rice. Avr-Pita enhances COX activity and decreases ROS accumulation in the host cell, and suppresses host innate immunity by perturbing ROS metabolism in the mitochondria [120]. 

Pi54 (earlier Pi-kh) is a dominant R gene that encodes ~43 kD protein and has a unique Zinc finger domain that overlaps with the leucine-rich repeat regions and belongs to the NBS-LRR family of R protein [121]. Unlike Pita, Pi54 is induced only in response to pathogen attack [121,122]. In *Magnaporthe oryzae* AVR-Pi54 gene encodes a predicted secreted protein with a signal peptide (SP) at the N-terminal region. The molecular docking study revealed that AVR-Pi54 protein physically interacts with Pi54 protein through novel non-LRR domains such as STI1 and RhoGEF. The STI1 and GEF domains that interact with AVR-Pi54 are also components of the rice defense complex [28,123]. Microarray analysis in transgenic rice performed at 72 h post-inoculation of the *Magnoporthe oryzae* revealed that many defense-related genes, such as PAL, laccase, callose, peroxidase, and enzymatic activities of defense response enzymes viz., phenylalanine ammonia-lyase, polyphenol oxidase, b-1,3-glucanase, peroxidase, chitinase, and b-glucosidase, were significantly Up-regulated [124]. 

Pik is a major R gene located on the long arm of chromosome 11 of rice and requires two NRL receptors, Pik-1 and Pik-2, to trigger cell death upon binding to the AVR-PikD [125]. Effector AVR-PikD interacts with specific rice HMA domain-containing heavy metal-associated isoprenylated plant proteins (OsHIPP19) and heavy metal-associated plant proteins (HPPs) [125]. Both Pik-1 and Pik-2 belong to the coiled-coil nucleotide-binding site leucine-rich repeat (CC-NBS-LRR) class of R proteins [126]. AVR-Pik encodes a secreted protein with a signal peptide at the N-terminus. Pikh-2 initiates host defense response and also physically interacts with the CC domain of Pikh-1 directly. AVRPik-D and Pikh-2 both bind the CC domain of Pikh-1 and form a complex AVR-Pik-Pikh-1-Pikh-2, then a specific signal is transferred from AVR-Pik to Pikh-2 and mediates resistance responses in Rice [83,127]. 

Pia gene encodes R protein present on chromosome 11 of O. sativa. Pia is composed of two adjacent NLR protein genes, RGA4 and RGA5, and is required for Pia and AVR-Pia interaction [82,128,129]. AVR-Pia encodes a predicted secreted protein with an SP at the N-terminus [81,130]. RGA5 transcripts generate two isoforms by alternative splicing, RGA5-A and RGA5-B. Only RGA5-A is required for Pia-mediated resistance. RGA4 acts as a constitutively active cell death inducer and is inhibited by RGA5 in rice plants without pathogen infection. However, RGA5 is an Avr receptor and has no role in cell death induction. RGA5 and RGA4 form hetero-complexes, and when AVR-Pia or AVR1-CO39 physically binds to the C terminal, non- LRR domain of RGA5, the interaction releases RGA4 and induces hypersensitive cell death in O. sativa [82,129]. 

AVR-Pii encodes a secreted protein belonging to the pex33 protein family, having four homologs [81]. AVR-Pii first accumulates in the BIC and is then translocated into the host cytoplasm [131]. Avr-Pii form complex with OsExo70-F2 and OsExo70-F3. OsExo70-F2 and OsExo70-F3 are presumably involved in exocytosis, and these proteins stably form homo- and hetero-dimers that incorporate AVR-Pii. R gene Pii encodes a 1025-amino acid protein predicted to be an NLR protein [132]. R protein Pii and AVR-Pii interact indirectly in the host cell [133]. In the absence of R protein Pii, AVR-Pii directly binds to Os-NADP-ME2 (Os nicotinamide adenine dinucleotide phosphate-malic enzyme), inhibits their activity, and thereby leads to the reduction of the PAMP-triggered ROS burst and successful biotrophy [131]. However, in the presence of Pii, OsExo70 acts as a decoy or helper in Pii/AVR-Pii interactions and activates defense response against *Magnoporthe oryzae* [133]. 

AvrPiz-t codes for a predicted secreted protein [80] and is first secreted into the BIC before translocation into the rice cell [77]. Effector AvrPiz-t targets 12 APIPs (AvrPiz-t interacting proteins) of rice. Among the twelve APIPs, APIP6 and APIP10 are functional ring E3 ubiquitin ligases, APIP5 is a bZip transcription factor, and APIP12 is a nucleoporin2 domain (Pfam 04096) containing protein [77,134,135]. Piz-t and AvrPiz-t interact indirectly [77,134,135]. Effector AvrPiz-t participates in both PTI and ETI of rice to *Magnoporthe oryzae* and targets the rice RING E3 ubiquitin ligases APIP6 (AvrPiz-t Interacting Protein 6) and APIP10 by promoting their degradation to suppress PTI in rice [77,136]. APIP10 promotes the degradation of Piz-t via ubiquitination. AvrPiz-t can remove negative regulation of Piz-t by degradation of APIP10 through the AvrPiz-t/APIP10 protein–protein interaction [136]. At the necrotrophic stage, a functional bZip transcription factor, APIP5 interacts with AvrPiz-t directly. APIP5 form homo-dimers and then interact with AvrPiz-t through its bZip DNA-binding domain at the N-terminus. The interaction suppresses APIP5 transcriptional activity and protein accumulation, leading to cell death. Thus, AvrPiz-t promotes effector-triggered necrosis (ETN) in the absence of Piz-t. When Piz-t is present in the rice cell, the N-terminus of Piz-t also interacts with the N-terminus of APIP5 and stabilizes APIP5 accumulation and activity to prevent rice cell necrosis. At the same time, APIP5 promotes the accumulation of Piz-t to maintain its basal level for providing resistance [134].

## 6. Disease Screening Protocols for Blast Resistance in Rice

The first step in a resistance breeding program is to rapidly screen all the available genetic stocks, including the local land races, improved cultivars, and exotic germplasms, using empirical techniques in glass houses or by field tests. Efficient, accurate, and reliable methods for screening disease resistance/susceptibility in crop plants are very important in developing resistant crop varieties in a relatively short period of time in a sustainable manner [137]. Based on the accuracy of the screening methods, resistant or susceptible genotypes might be identified for further breeding programs. Standard screening protocols of rice varieties for susceptibility to rice blast are usually carried out by spraying the plant with conidial suspensions under greenhouse and field conditions using local isolates [138]. Details of individual techniques are briefly described hereunder.

### 6.1. Field Screening Technique

**Figure 3:**
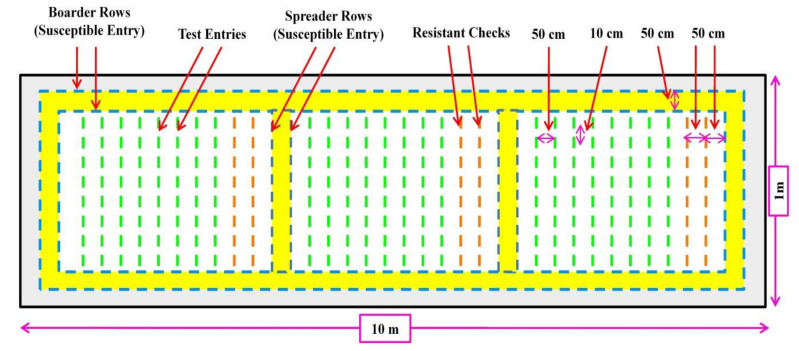
Schematic representation of Uniform Blast Nursery for screening of rice genotypes for blast disease.

A high-throughput and reliable field screening protocol for rice blast resistance are essential for the identification of resistant germplasm/varieties and resistant genes for further breeding programs [139]. In field conditions, artificial leaf blast disease screening usually takes place in a Uniform Blast Nursery (UBN) [138,140,141], which has a 10 m length and 1 m width (Figure 3). Test entries (30 plants/test entry) are planted in a nursery bed at a spacing of 10 cm plant to plant and 50 cm row to row. Moreover, a mixture of susceptible check entries is planted after every 10 lines of test entry as a spreader line and also planted throughout the border of UBN as a border line to facilitate the even spread of the blast disease. One or more susceptible entries could be used as border/spreader lines. The soil in the UBN is enriched with farm yard manures (FYM) and recommended doses of fertilizers. However, applying an excess rate of nitrogen fertilizer (150 kg N/ha) makes rice more vulnerable to spreading blast infection [138]. Simultaneously, isolation, maintenance, and multiplication of local and highly virulent blast cultures (fungal conidia) should be performed according to the method suggested by Vasudevan et al. [141], Prasad et al. [142], and Chhallagulla et al. [143]. Moreover, artificial inoculation is performed with a local and highly virulent blast race (fungal conidial suspension at a concentration of 1 × 10^5^ spores/mL) by spraying on UBN beds at 25–30 days after sowing (DAS). Later, the nursery beds are water sprayed 3–4 times per day and are covered with polythene sheets during the night to maintain a high humidity until disease development and progression are observed in border lines and spreader lines [138,141,143]. The observations on disease resistance or susceptibility are taken from each entry 10–15 days after the artificial inoculation and taken 2–3 times at 5 days intervals using the Standard Evaluation System 2002 (SES) of International Rice Research Institute, Manila, Philippines [144]. In this system, the disease score has ranged from 0–9 based on the severity of blast infestation in the leaves of the plant. The lines with disease scores of 6–9 are considered susceptible lines, 4–5 as moderately resistant, and 0–3 as highly resistant [144].

Several studies have been reported for using standard blast screening techniques and identified a number of novel resistance sources and genes. Vasudevan et al. [141] conducted a large-scale screening of 4246 geographically diverse rice accessions originating from 13 major rice-growing countries to identify a new resistance source for blast resistance. These accessions were selected from over 120,000 accessions based on their annotated rice blast resistance information in the international rice gene bank. The rice lines were screened using a two-step screening protocol which includes natural infection in a rice uniform blast nursery followed by artificial infections with five single rice blast isolates. Systematic screening for rice blast resistance was performed both under field and controlled environmental conditions, and rice cultivars IR72 and CO39 were used as susceptible control lines. Among the lines screened, 289 accessions showed broad-spectrum resistance (BSR) against all five single rice blast isolates. The accessions showing BSR were genotyped for the presence of the Pi2 resistance gene for the identification of promising accessions for the isolation of allelic variants of the resistance gene. Blast monogenic resistant lines for Pi54 (IRBLkh-K3), Piz-t (IRBLzt-T), Pi9 (IRBL9-W), Pita (IRBLta-CP1), and Pi2 (IRBLz5-CA), Pib (IRBLb-B) were used as control lines to isolate specific rice blast resistance genes. 

Similarly, Qin et al. [139] developed a high-throughput and reliable blast resistance evaluation system at the field level for the breeding of resistant varieties. This method consists of the following steps (i) pretreatment of diseased straw; (ii) sowing of seeds (iii) initiating seedling blast of the first batch of spreader population (iv) Sowing of seeds (v) and inducing seedling blast of the second batch of spreader population and test materials (vi) induction of seedling blast. Based on this protocol, a total of 730 indica hybrid rice were screened. This procedure enables uniform and consistent infection, which facilitates efficient and accurate assessment of seedling blast resistance for diverse rice materials.

### 6.2. Screening Techniques under Greenhouse/Polyhouse/Controlled Conditions 

Under a greenhouse/polyhouse screening procedure, the test entries 15 plants/test entry) are sown in plastic trays (10 rows × 2 columns per tray) in 4–5 batches for inoculation with different individual blast isolates (The number of batches can be increased/decreased based on the availability of individual isolates of the pathogen for screening). Plants are allowed to grow in normal conditions for 10–15 days. Rice blast pathogen cultured on culture medium may be taken for preparation of conidial suspension at 1 × 10^5^ conidia/mL of water. All the plants are then inoculated with 50 mL of spore suspension solution with a concentration of 1 × 10^5^ spores/mL with 1% tween-20 per tray after 10–15 DAS [141,145]. 

After inoculation, the plants need to be kept in a moist chamber at 26–28 °C for 24 h to maintain temperature and humidity. Plants should be transferred to the incubation chamber at 25 °C ± 2 for 1 week, and water is sprayed three to four times during day time to maintain high humidity (humidity should be near 100% for the initial 72 h to favor disease initiation [141,145]. Then the disease reaction should be assessed after nine days of inoculation and scored on a zero to nine rating scale as per the Standard Evaluation System 2002 (SES) of the International Rice Research Institute. Manila, Philippines [144]. 

### 6.3. Molecular-Marker-Based Screening of Rice Genotypes for Blast Resistance

In addition to the phenotypic screening for the identification of blast resistance source, molecular markers linked to the blast resistance genes/genes-specific primers are being utilized to identify the presence of resistance genes in the rice germplasm/line [146]. Tightly linked molecular markers, viz., simple sequence repeats (SSRs), single nucleotide polymorphisms (SNPs), and CAPSs (Cleaved Amplified Polymorphic Sequences), have been widely used for screening rice genotypes for blast-resistant genes [22,147,148,149,150,151,152,153]. Molecular-marker-based techniques not only allow the identification of resistant lines in a non-destructive manner but also help in the identification of broad-spectrum resistance genes and alleles of different genes and a combination of resistance genes in the germplasm [149]. This method gives fast and accurate results within a short period of time. With the advent of molecular markers paved the path for changing the paradigm of rice breeding by identification of genomic regions controlling several economic traits and their deployment in the elite rice lines using MAS and MABB approaches. 

Singh et al. [149] screened rice accessions with allele-specific SSR markers to identify 10 major blast resistance genes (Piz-5, Pi-9, Pitp(t), Pi-1, Pi-5(t), Pi-33, Pi-b, Pi27(t), Pi-kh, and Pi-ta) in 192 rice germplasm accessions from different ecological regions. They found genetic frequencies of the 10 major rice blast resistance genes varied from 19.79% to 54.69%. Interestingly, they found 17 rice accessions that harbored seven to eight major blast resistance genes indicating their exploitation in further breeding programs for developing blast-resistant cultivars. Furthermore, Imam et al. [150], Shikari et al. [151], Yan et al. [152], and Teerasan et al. [153] also used the linked SSR markers for screening of blast resistance in rice genotypes. Kim et al. [147] employed eight SNP markers (tightly linked with six major genes, Piz, Piz-t, Pik, Pik-m, Pik-p, and Pit) to determine the genetic diversities of blast resistance (R) genes from 86 accessions of aromatic rice and found four accessions of indica type carrying the six major genes. Moreover, Kim et al. [148] used SSR and CAPS markers for blast resistance screening in rice genotypes.

## 7. Strategies to Develop Durable Resistance in Rice Genotypes against the Blast Disease

**Figure 4:**
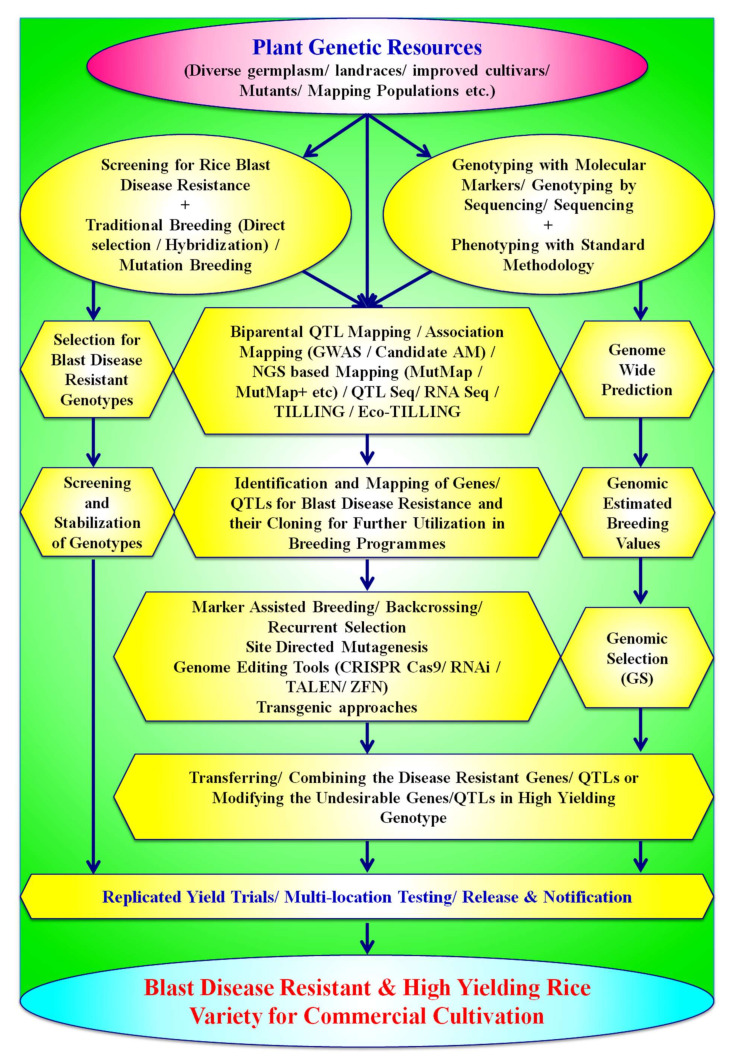
Schematic representation of various breeding biotechnological approaches used for development of blast-resistant rice varieties.

Many agronomical management practices, biological control, chemical control, disease forecasting, etc., have been adopted by the farmers to escape the hazards and reduce the losses caused by blast disease, but none of them has proven to be 100% efficient. Furthermore, the application of excessive fungicides to control the disease may cause severe loss of biodiversity, a threat to ecology, and produce hazardous food material [1,9,42]. Therefore, the development of broad-spectrum and durable resistance in rice varieties for blast disease is the only hope to combat the infestation of *Magnoporthe oryzae*. Thus, rice breeders should focus on developing durable, resistant rice varieties as resistance genes provide a worthwhile and environmentally safe option for the management of blast disease [19]. Many approaches, viz., traditional breeding methods, molecular-marker-based breeding approaches, transgenic breeding, genome editing-based methods, etc., are being deployed as major weapons to develop durable resistant varieties and are ecologically as well as economically sustainable. Advancement in genomic approaches and bioinformatic tools has led to the foundation for developing blast-resistant rice varieties with more accuracy and precision in a limited time period. A brief account of various breeding and biotechnological tools which can be used for the development of resistant rice varieties are briefly described in subsequent paragraphs and also presented in Figure 4.

### 7.1. Conventional Breeding Strategies for Developing Resistance against Rice Blast

The traditional breeding approaches are robust and oldest methods for developing novel genetic variants for blast resistance. Breeders have developed many blast-resistant rice varieties through traditional breeding methods, viz., pedigree method, backcross breeding, introduction and acclimatization, multiline breeding, pureline selection, recurrent selection, and mutation breeding. Interestingly, conventional breeding has enabled the International Rice Research Institute, Manila, Philippines, to generate elite cultivars with an enormous range of disease-resistant genes [12]. This approach is appropriate for developing durable and sustainable resistant rice varieties; however, its long and time-consuming breeding cycle and laborious nature are the major drawbacks of this method [19]. 

Introduction and acclimatization are the important, easiest, fastest, and most economically efficient breeding methods used to develop resistant varieties by introducing promising entries into new areas or regions where they have not been cultivated before. Most of the disease-resistant lines developed by IRRI, Manila, Philippines, have been disseminated to various rice-growing countries through the introduction and acclimatization process and used as breeding material for developing new disease-resistant varieties either through pureline selection or through the hybridization method [20]. Rice varieties IR36 and IR64 are the best examples of introduction and utilization in the development of new varieties. IR36 and IR64 contain the Pita gene, and IR64 also has another closely linked gene, Pi20, conferring resistance to blast disease. In addition to these known Pi genes, these varieties have accumulated several defense genes with their complex lineages [154,155], which make them durably resistant to blast disease in most locations. These varieties are used extensively in breeding programs in the southern states of India, where the blast is recognized as a potential threat to increased productivity [156]. 

Moreover, scientists from Indonesian Center for Rice Research (ICRR), Indonesia, have used more than 30 blast-resistant varieties of traditional and introduced materials for hybridization-based breeding programs. They have conducted the selection for blast resistance in a greenhouse through artificial inoculation of 19 pathogen races available in the ICRR’s collection. Interestingly, a total six promising blast-resistant lines, viz., TB490C-TB-1-2-1, TB361B-30-6-2, BP1976B-2-3-7-TB-1-1, TB356BTB-18-3, IR30176, and IR60080-23, with different patterns of resistance to the pathogen races were selected through participatory varietal selection and tested under farmers field [157].

Furthermore, multiline varieties or a mixture of several resistant near-isogenic lines can be used to reduce the outbreaks of blast disease. These lines carry resistance to different races of the same pathogen, which helps in developing durable resistance against the pathogen. Several scientists confirmed the use of multiline varieties for the control of the severity of blast disease [158,159,160,161,162]. The multiline variety ‘‘Sasanishiki’’ has been developed at the Furukawa Agricultural Experiment Research Station, Japan, in 1995 and commercially cultivated in 5,800 hectares of farmers’ fields in Miyagi Prefecture of northern Japan in 1997 as a blast-resistant rice variety. It consisted of seven different lines from BL1 to BL7, which carried seven different resistant genes against blast. These lines were developed by continuous backcrossing of Sasanishiki (recurrent parent) with blast-resistant land races of cultivars (Donor parent) [163]. Moreover, Zhu et al. [164] suggested using a cultivar mixture consisting of 80–90% resistant plants and 10–20% susceptible plants of similar varietal background to reduce the rapid evolution and emergence of new virulent *Magnoporthe oryzae* [160,161].

Simultaneously, the concept of shuttle breeding was also deployed during the 1970s and 1980s for developing blast-resistant rice varieties. Over 3000 rice germplasms were evaluated in 31 countries at 126 test sites from 1975 to 1992 under the International Network for Genetic Evaluation of Rice (INGER) [165,166]. A total of 522 resistant entries of the International Rice Blast Nursery (IRBN) were utilized in the hybridization program across 18 countries from 1984 to 1992 [166].

Furthermore, a few promising donors such as Ram Tulsi, Oryza nivara, Dawn, Tetep, Carreom, Zenith, Gam pai 15, Pankhari 203, and a number of improved plant types resulted from this study and were used on a regular basis as parents in blast-resistant breeding programs by various countries [165,166]. 

The pedigree method is the most efficient and extensively used breeding method for handling segregating generations from crosses, and a large number of varieties have been developed in crops such as rice, wheat, barley, sorghum, pulses, oilseeds, etc. It is the most appropriate method for improving the disease resistance ability of existing cultivars within a short span of time if the resistance is governed by major genes [20]. It is possible to combine genes for resistance to six or seven major diseases and insects in a short period through the pedigree method [12,39]. Most of the IRRI bred lines viz., IR24, IR34, IR36, IR60, IR56, IR64, IR46, and IR74 having blast-resistant genes have been used extensively in a breeding program for developing the blast-resistant rice varieties through the pedigree method [20,154]. Moreover, the TN-1 rice variety has also been used in breeding for blast resistance as a susceptible parent. Martínez et al. [167] have developed many blast-resistant genotypes from the segregating lines of Fanny (highly susceptible to blast) and 11 cultivars differing in blast resistance through the pedigree method. However, maintenance of accurate pedigree records and long breeding cycles are the major limiting factors for deploying this method. Moreover, this method will not be fruitful when blast resistance is governed by polygenes [12,19]. Backcross breeding is another widely used and common technique in rice breeding for transferring genes and chromosomes from one variety to another and from related species [20,168]. It has been extensively used for transferring disease resistance to popular and widely adapted varieties. For instance, a backcross was made between IR68835-98-2-B-2-1-1 (a broad-spectrum blast resistance variety) and KDML105 (a susceptible variety), and 83 lines from BC3F2 generation were evaluated for resistance against 12 different strains of the blast. All the BILs (Backcross Introgression Lines) displayed a low level of disease score. These lines can be further utilized in breeding programs for developing blast-resistant varieties [169]. Moreover, backcross breeding was integrated with molecular marker-based techniques to make the breeding process more clear, accurate, and authentic and also to reduce the exhaustive breeding exercise of maintaining a huge population. 

In addition to the above-mentioned methods, recurrent selection has also been deployed in rice breeding programs for the development of blast-resistant varieties [170]. Recurrent selection is characterized by being a cyclical method in which gains for the trait or traits under selection occur gradually and continuously. Interestingly, this method requires shorter breeding cycles and provides better genetic gains with wider genetic diversity in breeding lines for blast resistance. The durable blast-resistant rice variety CG-91 was developed through recurrent selection [171]. 

Conventional breeding methods have played a significant role in sustaining food production for burgeoning populations. However, conventional breeding takes more time and effort, which can be reduced nowadays due to advancements in genomic technologies. Conventional breeding and phenotyping are inefficient when several lineages of the pathogen are present, and resistances to multiple lineages are warranted [156]. Moreover, the success in resistance breeding is generally affected by linkage drag, due to which undesired traits closely linked with resistance genes are also transferred in new selections. Therefore, molecular-marker-based breeding approaches are now preferable in resistance breeding towards increasing its efficiency.

### 7.2. Mutation Breeding for Blast Resistance in Rice

A mutation is a rare event, reversible and recessive in nature, and a primary source of all genetic variations existing in any organism, including plants. Mutation breeding in rice is used to complement conventional breeding since this technique is very effective for improving one or a few traits, such as agronomic traits and resistance to pests and diseases, without altering the unique properties of improved variety/germplasm to make them easy acceptable among the farmers [172]. By the year 2022, about 3402 mutant varieties in more than 225 crops have been developed through induced mutagenesis by different countries and registered in the FAO/IAEA Mutant Variety Database (MVD), International Atomic Energy Agency (IAEA), Vienna, Austria [173] which has made significant contributions for food and nutritional security. 

Furthermore, many attempts have been made to develop disease resistance in rice against blast through mutation breeding. According to FAO/IAEA MVD [173], a total of 151 rice mutants having blast resistance have been developed across the World and registered in FAO/IAEA MVD. Interestingly, 72 rice mutants were developed through directly induced mutagenesis (Table 1), and 79 varieties were developed by crossing with mutant variety (Supplementary Table S1). Among the 72 rice mutants, four varieties were developed by chemical mutagenesis, whereas 68 were developed by the use of physical mutagens [173]. China, Japan, and India are the top three countries that have developed 56, 54, and 15 rice mutants, respectively [173]. The mutant variety Xiongyue 613 was the first officially approved blast-resistant rice variety, which was developed through mutation breeding (200 Gy of gamma rays) in 1965. The main improved attributes of Xiongyue 613 are moderate resistance to blast, higher yield, and good quality FAO/IAEA MVD, 2022). Interestingly, with the advancement in technologies, China has developed five rice mutants viz., Zhe 101, Hangtian 36, Huahang-simiao, Liangyouhang 2, and Neiyouhang 148 through the use of cosmic rays (treatment of seeds in aerospace). In India, a total of 15 rice mutant varieties that have blast resistance have been developed and registered in FAO/IAEA MVD, among which some mutants were developed through radiation-induced (X-rays, gamma rays) mutation breeding, while others were the results of hybridization with mutant variety. In India, ‘Jagannath’ is the first rice mutant produced in 1969 by X-rays irradiation of the popular tall variety T141, which was found to be resistant to blast [173]. Recently, a high-yielding and blast-resistant variety Vikram-TCR has been developed through radiation-induced mutation breeding under the joint collaboration of Indira Gandhi Krishi Vishwavidyalaya, Raipur, India, and Bhabha Atomic Research Centre, Mumbai, India. This variety has been developed with the help of gamma radiation (300 Gy) from the Safri-17 landrace, which is susceptible to blast disease [174,175]. When looking at the outcomes of the mutation breeding, it can be said that it is playing a significant role in combating the impact of blast disease in rice.

### 7.3. Molecular-Marker-Based Approaches for Resistance to Rice Blast Disease

Conventional breeding methods are slow and time-consuming, with uncertain results due to environmental impacts. With the advent in molecular techniques, a combination of traditional methods which are aided by new molecular techniques such as gene pyramiding, marker-assisted selection (MAS), marker-assisted back cross (MABC), QTL mapping, genome-wide association studies (GWAS), etc., can help accelerate the process of selection, screening, and development of blast-resistant rice varieties. Consequently, the identification and mapping of these R genes/alleles through advanced genomic approaches will be helpful in modern plant breeding for developing durable, resistant varieties [22,26,176]. Mining and characterization of disease-resistant genes/QTLs and their further deployment for developing resistant cultivars are the most preferred strategies by plant breeders. About 100 years ago, Sasaki [177] had, for the first time, reported the resistant varieties for rice blast fungus, *Magnoporthe oryzae*, in Japan, and Kiyosawa [178] identified the first rice blast gene Pi-a from a japonica rice variety “Aichi Asahi”. Interestingly, until now, about 146 R genes for rice blast resistance have been identified and mapped from both indica and japonica subspecies of rice by various scientists (Table 2). Among the 146 identified R genes, scientists have molecularly characterized and cloned 36 genes so far (Table 3) [15,24,42].

Furthermore, out of the 146 mapped R-genes, about 72% (105) are located on chromosomes 2, 6, 11, and 12 containing 13, 26, 38, and 28 genes, respectively. In addition, scientists have identified and mapped more than 500 QTLs for blast resistance in rice through linkage-based QTL mapping and Genome Wide Association Mapping (GWAS), from which 23 blast resistance loci viz., PiGD-1(t), Pi25(t), Pi26(t), Pi27(t), Pitq1, Pitq5, Pitq6, Pizh, Pi24(t), Pi25(t), Pi28(t), Pi29(t), Pi30(t), Pi31(t), Pi32(t), PiGD-3(t), Pi35(t), PiGD-2(t), Pilm2, Pi7(t), Pi34, and Pi21 have been isolated [15,24,176,179]. Among the 36 cloned R genes, the Pik locus is very important as it harbors a number of blast R genes (Pik, Pikm, Pikp, Piks, Pikh, and Pi1) which are being utilized regularly in rice breeding for developing durable resistance against the blast disease [176,180,181]. Several studies reported that R genes from the Pi2/9 locus of chromosome 6 had been extensively used in the breeding for blast resistance [182,183,184]. Similarly, the cultivar IR 64 contains 11 R-genes for blast resistance which are regularly utilized as donor parents in breeding for blast-resistant varieties. Interestingly, some of the major R-genes such as Pikh, Pi-1, Pi9, Pi20, Pi27, Pi39, Pi40, and Pit have broad-spectrum resistance (BSR) against the pathogen *Magnoporthe oryzae* [24,185].

**Table 2:** List of 146 blast-resistant genes identified and mapped in rice.

S. No.	Name of Blast-Resistant Genes	Name of Source Genotype	Reporting Year	Chromosome Number	Genomic Position (Mb)	Name of Linked Molecular Marker	Name of Country	References
1 *	Pb2	Jiangnanwan	2022	11	1.47	SNP	China	Yu et al. [15]
2	Pi67	Tetep	2019	12	12.09	SSR	India	Joshi et al. [186]
3	Pi57(t)	IL-E1454	2017	12	10.8	SSR, STS	Myanmar	Dong et al. [187]
4	Pi65(t)	Gangyu 129	2016	11	28.22	SNP, InDel	–	Zheng et al. [114]
5	Pi-jnw1	Jiangnanwan	2016	11	27.36	SSR, InDel	–	Wang et al. [188]
6	Pi66(t)	AS20-1	2016	3	26.78	SSR	Australia	Liang et al. [189]
7	Pita3(t)	IRBLta2-Re	2015	12	9.89	SSR	–	Chen et al. [190]
8 *	Pik-e	Xiangzao 143	2015	11	28	SSR, InDel	China	Chen et al. [190]
9	Pi-h2(t)	HR4	2015	1	7.9	SSR	India	Xiao et al. [191]
10	Pih3(t)	HR4	2015	12	12.95	SSR	India	Xiao et al. [191]
11	Pi-h1(t)	HR4	2015	11	28.11	SSR, InDel	India	Xiao et al. [191]
12 *	Pi64	Yangmaogu	2015	1	32.31	SSR, InDel	Japan	Ma et al. [192]
13	Pitb	Zixuan	2013	12	9.37	SSR, InDel	–	Sun et al. [193]
14	Pi61(t)	93-11	2013	12	9.98	InDel, SSR	China	Lei et al. [194]
15	Pi60(t)	93-11	2013	11	6.62	SSR, InDel	China	Lei et al. [194]
16	Pi58(t)	Haoru	2013	12	10.42	SSR	Myanmar	Koide et al. [195]
17	Pi51(t)	D69	2012	6	10.38	InDel, SSR	–	Xiao et al. [196]
18 *	Pi50(t)	EBZ, EBZ × LTH F2 and (EBZ × LTH) × LTH, BC1F2	2012	6	10.41	SSR, CAPS	–	Zhu et al. [197] and Jiang et al. [198]
19	Pi-hk1	Heikezijing	2012	11	27.66	SSR	–	Wu et al. [199] and Liu et al. [200]
20	Pihk2	Heikezijing	2012	9	10.17	SSR, InDel	–	He et al. [201]
21	Pias(t)	Asominori	2012	4	31.26	SSR, CAPS	China	Endo et al. [202]
22	Pi51(t)	Tianjingyeshengdao	2012	12	11.95	SSR, SFP	China	Wang et al. [203]
23	pi55(t)	Yuejingsimiao 2	2012	8	25.58	SSR, STS	China	He et al. [201]
24	Pi46(t)	H4	2011	11	27.74	SSR, InDel	–	Xiao et al. [204]
25	Pi-48	Xiangzi 3150	2011	12	11.95	SSR	China	Huang et al. [205]
26 *	Pi-a	Aichi Asahi	2011	11	6.49	SSR, InDel	Japan	Zeng et al. [206]
27	Pi-45(t)	Moroberekan	2011	4	31.49	SSR	Japan	Kim et al. [148]
28	Pi-42(t)	DHR9	2010	12	10.62	RAPD, SSR, STS	India	Kumar et al. [207]
29	Pi43(t)	Zhe733	2009	11	27.67	SSR	–	Lee et al. [208]
30	Pi-41	93-11	2009	12	16.74	SSR, STS	China	Yang et al. [209]
31 *	Pid3	Digu	2009	6	13.05	STS	China	Shang et al. [210]
32 *	Pik-p	K60, HR22	2009	11	28.05	SSR, CAPS	China	Wang et al. [211]
33	Pi2-2	Jefferson	2008	6	10.2	SSR	–	Jiang et al. [198] and Ballini et al. [179]
34 *	Pikahei- 1(t)	Kahei	2008	4	31.67	SSR, SNP	–	Xu et al. [212]
35 *	Pik-h	IRBLkh-K3, HP2216, and Tetep	2008	11	24.99	SNP	India	Xu et al. [213]
36	Pir2-3(t)	IR64	2008	2	–	SSR	Indonesia	Dwinita et al. [214]
37	Pirf2-1(t)	*O. rufipogon*	2008	2	–	SSR	Indonesia	Dwinita et al. [214]
38	Pi39(t)	Chubu 111, Q15	2007	4, 12	–	SSR	China	Liu et al. [215]
39	Pi-39(t)	Mineasahi and Chubu 111	2007	4	32.68	SSR	China	Liu et al. [215]
40	Pi-39	Q-15 and Chubu 111	2007	12	10.61	SSR	China	Liu et al. [215]
41	Pi-34	Chubu-32	2007	11	19.96	SSR	Japan	Zenbayashi et al. [216]
42	Pi-40(t)	IR65482, Co39, and O. australiensis (W)	2007	6	9.86	STS, SSR	Philippines	Jeung et al. [217]
43	Piz-5	C101A51_CO39	2006	6	–	–	–	Deng et al. [218]
44 *	Pi9	Cultivar TP309	2006	6	10.39	–	–	Qu et al. [219] and Koide et al. [195]
45 *	Pid2	Digu	2006	6	17.16	CAPS	China	Chen et al. [220]
46 *	Pigm(t)	Gumei 4	2006	6	10.36	CAPS, InDel	China	Deng et al. [218]
47	Pi51(t)	Tianjingyeshengdao	2006	12	–	–	China	Qu et al. [219]
48	Pi2-1	Tianjingyeshengdao	2006	6	10.08	SSR, SFP	China	Wang et al. [203] and Qu et al. [219]
49	Pi24(t)	Azuenca	2006	1	5.24	SSR	France	Nguyen et al. [221]
50	Pi-38	Tadukan	2006	11	22.48	SSR, AFLP	India	Gowda et al. [222]
51 *	Pi35(t)	Hokkai 188	2006	1	32.1	SSR	Japan	Nguyen et al. [221]
52 *	Pi-b	Tohoku, Koshihikari	2006	2	35.1	SNP	Japan	Hayashi et al. [223]
53 *	Piz-t	Toride No. 1	2006	6	10.39	STS	Japan	Zhou et al. [224]
54	Pi59(t)	Haoru_US-2	2006	6	10.82	SSR	Myanmar	Koide et al. [195] and Zhou et al. [224]
55	Pi-9(t)	IR31917	2006	6	10.38	STS	Philippines	Qu et al. [219]
56	Pi-Da(t)	Dacca 6	2005	2	2.21	SSR	–	Lei et al. [225]
57 *	Pi 37(t)	Cultivar St. No. 1	2005	1	33.1	SSR	China	Chen et al. [226]
58	Pi26(t)	Gumei 2	2005	6	11.06	RFLP, SSR	China	Wu et al. [227]
59 *	Pi-36(t)	Q61	2005	8	2.87	SSR, CRG	China	Liu et al. [228]
60 *	Pi54	Tetep	2005	11	25.26	SSR	India	Sharma et al. [121]
61	PiGD-2(t)	Sanhuangzhan 2	2004	10	–	SSR, RFLP, RGA	–	Liu et al. [229]
62	Pi-d1(t)	Digu	2004	2	34.94	SSR, RFLP	China	Chen et al. [230]
63	Pi-dt(2)	Digu	2004	6	17.16	SSR, RGA	China	Chen et al. [230]
64	Pig(t)	Guangchangzhan	2004	2	34.34	SSR	China	Zhou et al. [231]
65	Pi15	Q61 and GA25	2004	9	9.61	SSR, CRG	China	Liu et al. [229]
66	PiGD-1(t)	Sanhuangzhan 2	2004	8	16.37	SSR, RFLP, RGA	China	Liu et al. [229] and He et al. [201]
67	PiGD-3(t)	Sanhuangzhan 2	2004	12	14.45	SSR, RFLP, RGA	China	Liu et al. [229]
68	Pi-y2(t)	Yanxian No. 1	2004	2	35.03	SSR	China	Fukuta [232] and Lei et al. [225]
69	Pi-y1(t)	Yanxian No. 1	2004	2	35.03	SSR	China	Fukuta [232] and Lei et al. [225]
70	Pi27(t)	Q14 and Q61	2004	1	5.55	SSR	France	Zhu et al. [233]
71 *	Pi-tp(t)	CO39 and Tetep	2004	1	25.13	SSR	India	Barman et al. [234]
72	Pi-sh	Akihikari and Shin 2	2004	1	33.3	SSR	Japan	Fukuta [232]
73 *	Pik-s	Shin 2	2004	11	27.31	SSR	Japan	Fjellstrom et al. [235]
74	Pi28(t)	Azucena, IR64	2003	10	21.04	RFLP, RAPD	–	Sallaud et al. [236]
75 *	Pi56(t)	SHZ-2	2003	9	9.77	SSR, CRG, SNP	–	Jeon et al. [237]
76	Pizh	Zhai-Ya-Quing8	2003	8	4.38	–	China	Sallaud et al. [236]
77	Pi-25(t)	IR64	2003	2	34.36	QTL	France	Sallaud et al. [236] and Nguyen et al. [221]
78	Pi27(t)	IR64	2003	6	6.92	RFLP	France	Sallaud et al. [236]
79	Pi26(t)	IR64	2003	5	2.78	RFLP, RAPD	France	Sallaud et al. [236]
80	Pi-32(t)	IR64	2003	12	21.24	RFLP, RAPD	France	Sallaud et al. [236]
81	Pi-31(t)	IR64	2003	12	11.93	RFLP, RAPD,	France	Sallaud et al. [236]
82	Pi-29(t)	IR64	2003	8	13.93	RFLP, RAPD, Isozyme	France	Sallaud et al. [236] and Nguyen et al. [221]
83	Pi-30(t)	IR64	2003	11	4.41	RFLP, RAPD, Isozyme	France	Sallaud et al. [236] and Nguyen et al. [221]
84	Pi-33	IR64, Bala	2003	8	7.56	SSR, RFLP	France	Berruyer et al. [238] and Sallaud et al. [236]
85	Pii2	Ishikari Shiroke	2003	9	1.03	–	Japan	Pan et al. [239], Kinoshita and Kiyosawa [240]
86 *	Pi-5(t)	RIL249, Moroberekan	2003	9	9.77	AFLP, RFLP, CAPS	Philippines	Jeon et al. [237]
87 *	Pi2	5173, C101A51	2002	6	10.39	SSR, STS, RFLP	–	Jiang and Wang [241] and Zhou et al. [224]
88	Pi-24(t)	Zhong 156	2002	12	10.6	RFLP, RAPD, RGA	–	Zhuang et al. [242]
89 *	Pi-CO39(t)	Co39	2002	11	6.66	SSR, RFLP	USA	Chauhan et al. [243] and Huang et al. [205]
90 *	Pi25	Gumei 2	2001	6	18.09	–	China	Zhuang et al. [242]
91	Pi-25(t)	Gumei 2	2001	6	12.33	RFLP, RGA, SSR	China	Wu et al. [199] and Zhuang et al. [242]
92	PBR	St. No. 1	2001	11	–	RFLP, SSR	Japan	Fukuoka and Okuno [244]
93	Pi-47	Xiangzi 3150	2000	11	27.67	SSR	China	Huang et al. [205] and Ahn et al. [245]
94	Pi18	Suweon365	2000	11	28.93	RFLP	Korea	Ahn et al. [245]
95	Pi-lm2	Lemont, Teqing	2000	11	28.93	RFLP	USA	Tabien et al. [246]
96	Pi-tq5	Teqing	2000	2	34.61	RFLP	USA	Tabien et al. [246] Tabien et al. [247] and Zhou et al. [231]
97	Pi-tq1	Teqing	2000	6	29.02	RFLP	USA	Tabien et al. [246]
98	Pi-tq6	Teqing	2000	12	7.73	RFLP	USA	Tabien et al. [246]
99	Pi49	Mowanggu	1999	11	28.8	SSR	–	Sun et al. [193] and Chen et al. [248]
100	Pi-16(t)	AUS373	1999	2	34.94	RFLP, Isozyme	Japan	Pan et al. [249] and Zhou et al. [231]
101 *	Pb1	Modan	1999	11	21.71	–	Japan	Fujii et al. [250] and Hayashi et al. [251]
102	Pi-44(t)	Moroberekan	1999	11	28.93	RFLP, STS, AFLP	USA	Chen et al. [248] and Chauhan et al. [243]
103	Pi12	Hong Jiao Zhan K80-R-Hang Jiao-Zhan	1998	12	7.73	RFLP	Japan	Zhuang et al. [252]
104	Pi-19(t)	IRBL19-A and Aichi Asahi	1998	12	10.73	SSR	Japan	Koide et al. [195] and Hayashi et al. [253]
105	Pi-14(t)	Maowangu	1998	2	34.94	RFLP, Isozyme	Japan	Pan et al. [254] and Zhou et al. [231]
106 *	Pi3(t)	Pai-kan-tao	1997	9	7.8	–	–	Kinoshita and Kiyosawa [240]
107 *	pi-21	Owarihatamochi	1997	4	19.81	RFLP, SSR	Japan	Fukuoka and Okuno [244], Ahn et al. [255], and Pan et al. [254]
108	Pita-2	Yashiromochi, Pi No. 4	1997	12	10.6	RFLP, RAPD, SNP	Japan	Hayashi et al. [223]
109	Pi22	Suweon 365	1997	6	4.89	RFLP	Korea	Ahn et al. [255], Terashima et al. [256]
110	Pi23	Suweon 365	1997	5	10.75	RFLP, SSR	Korea	Ahn et al. [255], Rybka et al. [257]
111	Pi-20(t)	IR64	1997	12	12.95	SSR	Philippines	Li et al. [258] and Imbe et al. [259]
112 *	Pita	Tadukan, Yashiromochi	1997	12	10.6	RFLP, RAPD, SNP	USA	Rybka et al. [257], Hayashi et al. [223] and Bryan et al. [119]
113 *	Pi-k	Kusabue, Kanto 51	1996	11	28.01	RFLP, InDel, SNP	China	Hayasaka et al. [260] and Hayashi et al. [223]
114 *	Pik-m	Tohoku IL4, Tsuyuake	1996	11	28	RFLP, SSR	China	Kaji and Ogawa [261]
115	Pi157	Moroberekan	1996	12	12.37	RFLP	India	Naqvi et al. [262]
116 *	Pii1	Fujisaka 5	1996	6	2.29	–	Japan	Pan et al. [263]
117	Pikg	GA20	1996	11	27.31	–	Japan	Pan et al. [263]
118 *	Pit	K-59, Tjahaja, K-59	1996	1	2.27	RFLP, SNP	Japan	Kaji and Ogawa [261] and Hayashi et al. [223]
119	Pi8	Kasalath	1996	6	11.36	leucine aminopeptidase, phosphoglucose isomerase, RFLP	Japan	Pan et al. [263]
120	Pi62(t)	Yashiromochi	1996	12	7.73	RAPD, RFLP	Japan	Wu et al. [264]
121	Pi62(t)	Yashiromochi	1996	12	7.73	RAPD, RFLP	Japan	Wu et al. [264]
122	Pi-17(t)	DJ 123	1996	7	22.25	leucine aminopeptidase, phosphoglucose isomerase	Philippines	Pan et al. [263] and Zhu et al. [197]
123	Pib2	Lemont	1996	11	26.79	–	Philippines	Tabien et al. [265] (1996)
124	Pitq3	Teqing	1996	3		–	USA	Tabien et al. [265]
125	Pitq2	Teqing	1996	2	–	USA	USA	Tabien et al. [265]
126	Pitq4	Teqing	1996	4		USA	USA	Tabien et al. [265]
127	Pik-l	Liziangxintuanheigu, Kusabue	1995	11	27.69	SSR, STS, CAPS	China	Hayasaka et al. [260] and Hayashi et al. [223]
128	Pi-10(t)	Tongil	1995	5	14.52	RAPD	India	Naqvi et al. [265] and Wu et al. [227]
129 *	Pi-1(t)	Apura, C101LAC	1995	11	28	STS, RFLP, SSR, CAPS	USA	Parco [266], Yu et al. [267]
130	Pi(t)	P167	1994	4	2.27	–	–	Causse et al. [268]
131	Pi-11(t)	Zhai-Ye-Quing	1994	8	13.93	RFLP, RAPD	China	Causse et al. [268]
132	Pi-6(t)	Apura	1994	12	7.73	RFLP	USA	McCouch et al. [269]
133	Pi-7(t)	RIL29 (Moroberekan)	1994	11	18.64	12.37	USA	Wang et al. [270]
134	Pi26(t)	Azucena/Gumei 2	1993	5	2.07	–	France	Wu and Tanksley [271] Ahn et al. [255] and Nguyen et al. [221]
135	Pi-13	*O. minuta* (W), Kasalath	1992	6	15.83	SSR	Philippines	Amante- Bordeos et al. [272]
136	Pi3(t)	Pai-kan-tao	1992	6	–	–	Philippines	Mackill and Bonman [273]
137	Pi1	LAC23	1991	11	26.49	RFLP	Philippines	Yu et al. [274]
138	Pikur2	Kuroka	1988	11	2.84	–	Japan	Goto [275]
139 *	Pish	Nipponbare	1985	11	33.38	–	Japan	Imbe and Matsumoto [276]
140	Mpiz	Zenith	1976	11	4.07	–	Japan	Goto [277]
141	Piz	Zenith, Fukunishiki, Toride 1, Tadukan	1976	6	10.39	STS	Japan	Goto [277] and Zhou et al. [224]
142	Pif	Chugoku 31-1	1971	11	24.69	–	Japan	Shinoda et al. [278]
143	Pii	Ishikari Shiroke	1971	9	2.29	–	Japan	Ise [279] and Shinoda et al. [278]
144	Piis1	Imochi Shirazu	1970	11	2.84	–	Japan	Goto [280]
145	Pikur 1	Kuroka	1970	4	24.61	Isozyme	Japan	Fukuoka et al. [281] and Goto [280]
146	Pise	Sensho	1970	11	5.74	–	Japan	Goto [280]

‘-‘ Indicates the non-availability of data, * Indicates that the particular gene has been cloned and molecularly characterized.

**Table 3:** List of blast resistance genes cloned, cloning strategy and their location on rice chromosome.

S. No.	Name of Blast Resistance Genes	Proteins Encoded by R Genes	Donor Rice Lines/Genotypes	Chromosome No.	Year of Cloning	Cloning Approach Used for Isolation of R Genes	Reference Serial No. of Table 2	Reference
1	*Pb2*	NBS-LRR protein with NB-ARC domain and LRR domain	Jiangnanwan	11	2022	Map-based cloning	1	Yu et al. [15]
2	*Pid3-I1*	CC-NBS-LRR	MC276	6	2019	Gene Mapping	31	Inukai et al. [282]
3	*Pitr*	A typical protein with an armadillo repeat (Putative E3 ligase)	Katy	12	2018	Map-based cloning	71	Zhao et al. [283]
4	*Pigm*	NBS-LRR	Gumei 4	6	2017	Map-based cloning	46	Deng et al. [35]
5	*Pi64*	CC–NBS–LRR	Yangmaogu	1	2015	Map-based cloning	12	Ma et al. [192]
6	*Pi50*	NBS-LRR	Er-Ba-zhan (EBZ)	6	2015	-	18	Su et al. [113]
7	*Pik-e*	CC-NBS-LRR	Xiangzao 143	11	2015	Map-based cloning	8	Chen et al. [190]
8	*Pi35*	NBS-LRR	Hokkai-188	1	2014	Map-based cloning	51	Fukuoka et al. [284]
9	*Pi63/Pikahei-1(t)*	NBS-LRR	Kahei	4	2014	Map-based cloning	34	Xu et al. [285]
10	PiK-h	NBS-LRR	K3	11	2014	Positional cloning	35	Zhai et al. [127]
11	*Pi54of*	NBS–LRR	*Oryza officinalis* (nrcpb004)	11	2014	Map-based cloning	60	Devanna et al. [28]
12	*Pii*	NBS-LRR	Hitomebore	9	2013	MutMap-Gap	116	Takagi et al. [132]
13	*Pi-CO39*	CC-NBS-LRR	CO39	11	2013	--	89	Cesari et al. [82]
14	*Pi56*	NBS–LRR	Sanhuangzhan No. 2	9	2012	Map-based cloning	75	Liu et al. [200]
15	*Pi1*	CC–NBS–LRR	C101LAC	11	2012	Map-based cloning	129	Hua et al. [286]
16	*Pi54rh*	NBS-LRR	Oryza rhizomatis (nrcpb 002)	11	2012	Map-based cloning	-	Das et al. [287]
17	*Pi25*	CC-NBS-LRR	Gumei2	6	2011	Map-based cloning	10	Chen et al. [288]
18	*Pia*	CC-NBS-LRR	Aichi Asahi	11	2011	MB and mutant screening	26	Okuyama et al. [128]
19	*Pik-p*	CC-NBS-LRR	K60	11	2011	Map-based cloning	32	Yuan et al. [289]
20	*Pik*	CC-NBS-LRR	Kusabue	11	2011	Map-based cloning	113	Zhai et al. [180]
21	*Pish*	NBS–LRR	Shin-2	1	2010	Mutant Screening	139	Takahashi et al. [290]
22	*Pb1*	CC-NBS-LRR	Modan	11	2010	Map-based cloning	101	Hayashi et al. [251]
23	*Pi54/Pi-kh*	NBS-LRR	Tetep	11	2010	Map-based cloning	73	Sharma et al. [122]
24	*Pit*	CC-NBS-LRR	K59	1	2009	Map-based cloning	118	Hayashi and Yoshida [291]
25	*pi21*	Proline-rich heavy metal binding protein	Owarihatamochi	4	2009	Map-based cloning	107	Fukuoka et al. [281]
26	*Pi-d3*	CC-NBS-LRR	Digu	6	2009	In silico analysis	106	Shang et al. [210]
27	*Pi5*	CC-NBS-LRR	Moroberekan	9	2009	Map-based cloning	86	Lee et al. [292]
28	*Pik-m*	NBS-LRR	Tsuyuake	11	2008	Map-based cloning	114	Ashikawa et al. [293]
29	*Pi37*	NBS-LRR	St. No. 1	1	2007	Map-based cloning	57	Lin et al. [294]
30	*Pi36*	CC-NBS-LRR	Q61	8	2007	Map-based cloning	59	Liu et al. [215]
31	*Pi-d2*	B-lectin receptor kinase	Digu	6	2006	Map-based cloning	45	Chen et al. [220]
32	*Pi9*	NBS-LRR	75-1-127	6	2006	Map-based cloning	44	Qu et al. [219]
33	*Pi-2*	NBS-LRR	C101A51	6	2006	Map-based cloning	87	Zhou et al. [224]
34	*Piz-t*	NBS-LRR	Toride 1	6	2006	Map-based cloning	53	Zhou et al. [224]
35	*Pi-ta*	NBS-LRR	Yashiro-mochi	12	2000	Map-based cloning	112	Bryan et al. [119]
36	*Pib*	NBS-LRR	Tohoku IL9	2	1999	Map-based cloning	52	Wang et al. [295]

‘-‘ Indicates the non-availability of data.

Identification and mapping of the blast resistance R-genes through advanced biotechnological and genomic approaches are essential for their efficient utilization in molecular breeding programs, especially in marker-assisted selection and marker-based gene pyramiding of two or more R-genes for achieving the broad-spectrum and durable resistance [15,22,24,296,297]. In addition, this also gives information about the gene-linked, gene-based, and functional markers, which can enhance the efficiency of conventional resistance breeding programs. Further, this provides an opportunity for molecular characterization and cloning of R-genes [298]. Recent mapping studies, viz., linkage-based QTL mapping and linkage-disequilibrium-based association mapping, are the most widely used methods for identification and mapping of the blast resistance R-genes and have successfully illustrated the acceleration of the breeding program. Linkage-based QTL mapping was proposed as a useful molecular breeding technique for detecting the QTLs as it utilizes the biparental mapping population developed from two contrasting genotypes. In the past few years, more than 350 QTLs and blast resistance genes have been identified and localized on rice chromosomes for blast resistance through a linkage-based QTL mapping strategy [236,244,247,299,300,301,302,303,304,305,306,307,308,309,310,311,312]. 

In association mapping, genome-wide association studies (GWAS) detect the genetic variation (marker) polymorphisms of multiple individuals in the whole genome to obtain the genotype associated with the observable traits. In comparison with the linkage-based QTL mapping, it has the ability to map genes with high resolution, cover rich-captured variations, and have high efficiency in locating multiple traits simultaneously. GWAS has been deployed by scientists for the last two decades for the identification and mapping of blast-resistant genes and revealed more than 230 blast-resistant loci in rice which are distributed throughout the genome [15,22,115,176,313,314,315,316,317,318,319,320,321,322,323,324,325,326]. Among them, five rice blast resistance loci, including the cloned gene Pita, were first identified by GWAS [115,313]. 

The identified and mapped genes/QTLs could be easily deployed in the breeding program with the help of molecular markers. Hybridization, backcrossing, marker-assisted selections, and marker-assisted backcross breeding (MABB) are the most popular methods for the introgression of resistance (R) genes into the elite cultivar for improving the rice line for disease resistance. Gene pyramiding involves transferring more than one favorable gene/QTL of traits from multiple parents into a single genotype by marker-assisted selection (MAS). A number of studies have been made to deploy R genes in rice breeding programs for blast resistance through the gene introgression or pyramiding approach [27,185,327,328,329,330,331]. A series of improved intermediate materials with various blast resistance gene combinations or improved new varieties were bred to achieve broader and more durable resistance. The detail of rice lines/varieties improved by introgression of single, multiple genes for blast resistance and introgression of genes for multiple biotic stresses and a combination of biotic and abiotic stresses is presented in Table 4. 

Most studies focused on pyramiding individual resistance genes to develop single or multiple resistances [185,332,333,334,335,336,337,338], but these lines have less durability of resistance. Hence, a pyramiding of genes for a trait by combining two R-genes [182,327,339,340,341,342,343,344,345] or more than two complementary R-genes [230,343,346,347,348,349,350] could provide superior phenotypic benefits and stability compared to a single gene. Furthermore, looking at the current scenario of changing climatic conditions, breeding rice cultivars with durable multiple biotic stresses resistance through gene pyramiding will be the most effective method, and several scientists have already started to work on this aspect [27,331,349,351,352,353,354,355]. Hence, in this way, molecular markers could be very useful in breeding multiple disease resistance along with resistance in rice.

**Table 4:** List of blast resistance genes deployed for improvement of rice lines using marker-assisted selection (MAS) and marker-assisted backcross breeding (MABB).

S. No.	Gene/QTL	Trait/Resistance	Type of Molecular Marker	Technique/Approach Used	Application/Lines Developed	References
**Single gene for blast resistance**						
1.	*Pi1*	Resistance to blast disease	ISSR and SSR	MABB	Marker-assisted backcross breeding for improvement of variety Zhenshan97 A	Liu et al. [185]
2.	*Piz*	Resistance to blast disease	SSR	MAS	Tightly linked markers with *Pi-z* locus was applied for screening of germplasm for blast resistance in rice	Fjellstrom et al. [332]
3.	*Pita*	Resistance to blast disease	Gene-specific to gene	MAS	Applied for detection of *Pita* gene in 141 rice germplasms and introduction of gene through advanced breeding approaches	Wang et al. [333]
4.	*Pi9*	Resistance to blast disease	Gene-specific	Marker aided selection	Introgressed Pi-9(t) resistance gene in the cultivar Luhui 17	Wen and Gao [334]
5.	*Pi39*	Resistance to blast disease	InDel	MABB	Introgressed into Chinese cultivar Q15	Hua et al. [335]
6.	*Pikh*	Resistance to blast disease	SSR	MABB	Improvement of Malaysian Cultivar, MR264 by Introgression of *Pikh* gene	Hasan et al. [336]
7.	*Pi40*	Resistance to blast disease	SSR	MABB	Introgressed into elite cultivars Turkish, Halilbey and Osmancik-97	Beser et al. [337]
8.	*Pi-ar*	Resistance to blast disease	RAPD	MAS	Introgression of *Pi-ar* gene using double haploid technique	Araujo et al. [338]
**Two genes for Blast resistance**						
9.	*Piz-5*, *Pi54*	Resistance to blast disease	SSR	MABB	Blast disease resistance genes transferred to develop Pusa 1602 and Pusa 1603	Singh et al. [356]
10.	*Pi1*, *Piz*	Resistance to blast disease	SSR	MABB	Pyramiding of *Pi1* and *Piz-5* genes into PRR78	Gouda et al. [340]
11.	*Pi1*, *Pi2*	Resistance to blast disease	SSR	MABB	Introgressed into Intan variety and BPT5204	Hegde et al. [344]
12.	*Pi46*, *Pita*	Resistance to blast disease	SSR	MABB	Introgression of resistance genes into Hang hui 179 (HH179)	Xiao et al. [343]
13.	*Pi2*, *Pi9*	Resistance to blast disease	SNP	MABB	Introgression of blast resistance genes into R179	Luo et al. [345]
14.	*Pi-b* and *Pik-h*	Resistance to blast disease	SSR, RM 208, RM 206	MABB	Pyramided two blast resistance genes into MR219Malaysian rice variety	Tanweer et al. [327]
15.	*Piz-5* and *Pi54*	Resistance to blast disease	SSR	MABB	Incorporation of blast resistance into “PRR78”, an elite Basmati rice restorer line	Singh et al. [297]
16.	*Pi-2* and *Pi-54*	Resistance to blast disease	SSR	MABB	Introgression of blast resistance genes into the genetic background of elite, bacterial blight resistant indica rice variety, Improved Samba Mahsuri	Madhavi et al. [342]
17.	*Pi54* and *Pi1*	Resistance to blast disease	SSR	MAS	Introgression of blast resistance genes into cold tolerant variety Tellahamsa	Oddin et al. [341]
18.	*Pi46* and *Pita*	Resistance to blast disease	SSR	MABB	Blast resistance genes were introgressed into an elite restorer line Hang-Hui-179 (HH179)	Xiao et al. [343]
**More than Two genes for Blast resistance**						
19.	*Pi1, Piz-5, Pita*	Resistance to blast disease	RFLP	MAS	Pyramiding of three NILs namely (C101LAC, C101A51 and C101PKT) for blast resistance into a single cultivar CO39, each linecarrying resistance genes *Pi1, Piz-5* and *Pita*, respectively.	Korinsak et al. [169]
20.	*Pi1*, *Pi2*, *Pi33*	Resistance to blast disease	SSR	MABB	Introgression of multiple blast disease resistance genes into Jin23B	Chen et al. [302]
21.	*Pi1*, *Pi2*, *Pi33*	Resistance to blast disease	SSR	MAS	Improvement of Russian rice varieties by pyramiding of blast disease resistance genes	Usatov et al. [357]
22.	*Pi9*, *Pizt*, *Pi54*	Resistance to blast disease	SNP	MABB	Introgression of *Pi9*, *Pizt*, *Pi54* blast resistance genes into japonica rice 07GY31	Xiao et al. [343]
23.	*Pi1,Pi2*, *Pi33*	Resistance to blast disease	SSR	MABB	Improving blast resistance in Indian rice variety ADT43 by pyramiding three blast resistance genes	Divya et al. [346]
24.	*Pid1*, *Pib*, *Pita*, *Pi2*	Resistance to blast disease	SSR	MAS	*Pid1*, *Pib* and *Pita* genes were introduced into G46B, while *Pi2* was introduced into Zhenshan 97B	Chen et al. [230]
25.	*Pizt*, *Pi2*, *Pigm*, *Pi40*, *Pi9*, *Piz*	Resistance to blast disease	SSR	MAS	Introgression of multiple blast resistance genes into Yangdao 6	Wu et al. [350]
**Multiple stress tolerance**						
26.	*Xa21*, *Piz*	Resistance to blast and bacterial leaf blight disease	SSR	MAS	Introgression of Blast and Bacterial leaf blight disease resistance gene	Narayanan et al. [351]
27.	*Pi2* and *Xa23*	Resistance to blast and bacterial leaf blight disease	SSR	MAS	Introgression of broad-spectrum disease resistance genes into, elite thermo-sensitive genic male-sterile rice line-GZ63-4S	Jiang et al. [182]
28.	*Xa21* and *Pi54*	Resistance to blast and bacterial leaf blight disease	SSR	MABB	Introgression of BLB and blast resistance into DRR17B, an elite, fine-grain type maintainer line of rice	Balachiranjeevi et al. [348]
29.	*Pi1*, *Pi2*, *Xa23*	Resistance to blast and bacterial leaf blight disease	SSR	MABB	Introgression of bacterial blight and blast resistance into variety Rongfeng B	Fu et al. [358]
30.	*Pi2*, *Xa21*, *Xa33*	Resistance to blast and bacterial leaf blight disease	SSR	MABB	Introgressed bacterial blight and blast diseases resistance genes into RPHR-1005	Kumar et al. [359]
31.	*Pi9* *Drought*	Resistance to blast and drought tolerance	Gene linked markers	MAS	Pi9 has been introgressed into different genetic backgrounds of cultivated varieties, such as indica cultivar Swarna + drought	Dixit et al. [331]
32.	*Pi2, Pi54, xa13* and *Xa21*	Resistance to blast and bacterial leaf blight disease	SSR	MABB	Introgressed of bacterial blight and blast diseases resistance genes for improving disease resistance traits in Basmati rice varieties	Ellur et al. [352]
33.	*Xa21,xa13* and *Pi54*	Resistance to blast and bacterial leaf blight disease	Gene-specific	MABB	Pyramiding of bacterial blight and blast diseases resistance into Indian rice variety MTU1010	ArunaKumari et al. [353]
34.	*Xa21, xa13* and *Pi54*	Resistance to blast and bacterial leaf blight disease	SSR	MAS	Improvement of Vallabh Basmati 22 by Introgression of *Xa21, xa13* genes for Bacterial Blight and *Pi54 for* Blast disease resistant genes	Srikanth et al. [349]
35.	*Xa21, Xa33, Pi2, Rf3* and *Rf4*	Resistance to blast and bacterial leaf blight disease	SSR	MAS	Marker-assisted improvement of the elite restorer line of rice, RPHR-1005 for resistance against diseases	Kumar et al. [360]
36.	*Xa 5* and 4 blast QTLs	Resistance to blast and bacterial leaf blight disease	SSR	MAS	Introgression of bacterial leaf blight and blast resistance genes into rice cultivar RD6	Pinta et al. [361]
37.	*Xa13*, *Xa21*, *Pi54*, *qSBR11*	Resistance to blast and sheath bight disease	SSR	MAS	Transfer of multiple disease resistance genes for bacterial blight, blast and sheath blight disease in rice	Singh et al. [297]
38.	*Pi54, qSBR11-1, qSBR11-2 and qSBR7-1*	Resistance to blast and sheath bight disease	SSR and QTLs	MABB	Introgression of multiple disease resistance genes into a maintainer of Basmati rice CMS line	Singh et al. [347]
39.	*Pi2, Pi9*, *Gm1, Gm4*, *Sub1*, and *Saltol*	Blast disease, Gall Midge Submergence and Salinity tolereance	SSR and gene linked markers	MAS	Pyramiding of genes/QTLs to confer resistance/tolerance to blast, gall Midge, submergence, and salinity in a released rice variety CRMAS2621-7-1 as Improved Lalat	Das and Rao [362]
40.	*Pi9, Xa4, xa5, xa13, Xa21, Bph3, Bph17, Gm4, Gm8 and qDTY1.1 and qDTY3.1*	Blast Bacterial leaf blight Brown planthopperGall midge and QTLs for drought tolerance	Gene based/linked markers	Marker-assisted forward breeding	MAS in combining tolerance to multiple biotic and abiotic stresses in Swarna + drought recurrent parent	Dixit et al. [331]
41.	*Pi9, Xa21, Gm8, qDTY1.1, qDTY2.2 and qDTY4.1*	Blast, Bacterial blight (BB), Gall midge (GM) and QTLSs drought tolerance	Gene based/linked markers	Marker-assisted forward(MAFB) and back cross (MABC) breeding	Introgressed in to Indian elite rice variety, Naveen	Janaki Ramayya et al. [354]
42.	*BPH3, BPH24, Pi2, Pi9, Pita, Pib, Xa21Pimh, and* badh2	Brown Plant hopperblast disease, bacterial blight and Aroma	SSR and gene linked markers	MAS	Brown planthopper (BPH), blast, and bacterial leaf blight (BLB) resistance and aroma genes into elite rice maintainers and restorers	Wang et al. [27]
43.	*Xa21, Pi54, Pup1*	BB resistance gene, the blast resistance gene, and low soil phosphorous tolerance QTL/gene,	Gene/QTL linked markers	Marker-assisted pedigree breeding	BB resistance gene, the blast resistance gene, and low soil phosphorous tolerance QTL/gene in to MTU 1010 (CottondoraSannalu)	LaxmiPrasanna et al. [355]

### 7.4. Transgenic Breeding for Rice Blast Resistance

The most significant advancement in the area of varietal development for disease resistance is the use of the techniques of genetic engineering to develop transgenic rice resistant to diseases. This approach is advantageous for introducing disease resistance into elite rice cultivars since transgenic plants can acquire a single desired trait without any alteration of the original genetic background. Several studies have been performed to confer the disease resistance in rice against the *Magnoporthe oryzae* [28,29,30,31,363,364,365,366,367,368,369,370], which are presented briefly in Table 5.

Using Agrobacterium-mediated transformation, Nishizawa et al. [363] reintroduced the chitinase gene in Japonica rice varieties Nipponbare and Koshihikari. Two genes, Cht-2, which accumulated chitinase intracellularly, and Cht-3, which generated chitinase extracellularly, were introduced. It was found that even if either of the genes was expressed in the transgenic plants, they showed enhanced resistance to blast fungus. Studies have demonstrated the expression of the Gns1 gene, which is responsible for hydrolyzing glycosidic bonds in cell walls of the Poaceae family. Gns1 gene was introduced in the plants with CaMV35S as its promoter. The over-expression of this gene led to the development of resistance against blast disease, but it also resulted in stunted growth of the plants. The use of this gene for blast resistance is limited as its constant expression results in poor root formation, diminutive growth, and development of certain brown specks [365].

Interestingly, a different approach to rice blast resistance was undertaken by transferring regulatory and structural genes of maize which were responsible for the production of flavonoids. Flavonoids are responsible for generating various levels of stress responses in plants. In this study, anthocyanin production was increased in Tp309, a japonica rice variety, by the transfer of the C2 gene of maize [364]. Transfer of this gene might have resulted in a mutation leading to the generation of CHS protein which provided resistance against blast disease [364]. Furthermore, Coca et al. [366] developed blast-resistant transgenic rice by transferring the ER-CecA gene from the giant silk moth Hyalophora cecropia. This gene was optimized to produce Cecropin A peptides in paddy, which are a member of antimicrobial protein families. This transformation did not account for any pathogenesis-related gene expression, which is a good indicator of the direct effect of a gene on the pathogen [366]. 

Another promising approach to rice blast fungus resistance was exhibited by transferring an antifreeze glycopeptide gene using Agrobacterium as a medium. This gene was overexpressed in transformed paddy plants, which were able to withstand low temperatures such as −1 °C up to 24 h. Transgenic and control plants were recovered and thawed. Interestingly, transgenic plants displayed fewer symptoms and more resistance against blast fungus as compared to other fungi. This implies that such pathogenesis-related proteins can be identified and used to generate resistance against blast [367]. Similarly, another host defense antimicrobial peptide, thanatin, was transferred into the rice. The transformants displayed a significant level of resistance against blast fungus [368]. Similarly, Chen et al. [369] transferred the Pi-d2 gene with the help of three vectors into nine lines of paddy to generate blast-resistant rice varieties. This gene exhibited broad-spectrum resistance against blast as it displays resistance against 39 strains. The selection was made using the production of crude toxins by fungus. Helliwell et al. [370] suggested that ethylene plays a major role in resistance against rice blasts. They have generated transgenic plants using ACS2 (1-aminocyclopropane-1-carboxylic acid synthase), which produces ethylene.

The transformed lines showed resistance against blast with little or no difference between the agronomic traits of transgenics and wild-type plants [370]. Devanna et al. [28] isolated the Pi54 gene from a wild species, Oryza officinalis, and it was renamed Pi54, which confers resistance against blast disease. Interestingly this study demonstrated that the Pi54 of the gene was structurally more stable and provided a higher level of resistance as compared to Pi54. This gene was transferred in two susceptible rice lines, IET16310 (indica) and TP309 (japonica), which transformed them into highly resistant strains against *Magnoporthe oryzae*. 

Moreover, Wang et al. [29] transferred MoHrip1 and MoHrip2 genes into rice through an Agrobacterium-tumefaciens-based method, which resulted in resistance to blast disease. The transgenic paddy plants constrained the growth of fungal hyphae and also had a high water-retention capacity. Furthermore, marker-free transgenic rice was generated using maize’s Ac/Ds transposon vectors carrying fluorescent protein (GFP) and red fluorescent protein (mCherry) genetic markers to generate marker-free transgenic plants. Pi21 gene was expressed in these transgenic plants to generate resistance against rice blasts. The transformed lines had good resistance against *Magnoporthe oryzae* [30]. Moreover, three Pi genes, viz., Pib, Pi25, and Pi54, were transferred together into two rice varieties, the indica variety Kasalath and the japonica variety Zhenghan 10. The transformed varieties exhibited a good level of resistance against blast pathogens, but this gene pyramiding came with its baggage of linkage drag and pleiotropic effects of these genes. The transgenic plants were impairing many gene transcriptions, which ultimately interrupted the normal development of the plants [31]. 

The genes mentioned in the above studies suggest a potential future for transgenic crops, which will stand against huge threats to the staple food of the world. The genes can be used to generate new varieties or can be used to create strong resistance barriers against major diseases such as blasts. Genes from other organisms or species can be used to achieve this goal without negatively affecting the desired agronomical traits.

### 7.5. Genome Editing Tools for Developing the Blast Resistance in Rice

The emergence of fatal strains of the rice blast disease is imminent, and the existing tools and available resistance genes might not be enough to cope with their pace. Genome editing tools have provided us with new pathways and have given new perspectives for developing blast-resistant rice varieties. The genes or crops can be targeted to direct their characteristics in a particular direction and to create novel variations [372]. Several methods allow insertion, deletion, mutation, or substitution of nucleotides or sequences of nucleotides in specific locations. This can be accomplished by genome editing using highly advanced genetic engineering tools [373], such as endonucleases which are divided into four categories: Zinc Finger Nucleases (ZFNs), Meganucleases (MNs), chemical nucleases [374] TALENs (Transcription Activator-Like Effector Nucleases) and CRISPR Cas9 (Clustered Regularly Interspaced Short Palindromic Repeats) are the latest addition [375].

#### 7.5.1. CRISPR Cas9 Based Resistance for Rice Blast

In recent years, there have been a lot of changes in the environment that directly or indirectly affect the growth of important crops. Insect and disease infestations have increased drastically, and constant evolution in the strains resistant to existing defense mechanisms has forced the scientific community to develop new and innovative methods to fight against these odds. Rice blast is one such disease that causes a lot of damage to the crop and is considered among the top 10 fungal diseases that could threaten global food security [17,376]. Biotechnology helped researchers to identify genes that can confer resistance to rice blasts in resilient varieties of rice. Recently developed techniques such as CRISPR Cas9 can be very useful in developing varieties that are resistant to the attack of *Magnaporthe oryzae*. CRISPR is a very effective sequence-specific nuclease (SSN) that targets particular genes and carries out efficient genome editing. 

Wang et al. [97] used CRISPR Cas9 to edit the OsERF922 gene in rice. They have induced mutations in the target sites using the C-ERF922 enzyme, which generated several insertions and deletions. Interestingly, these mutations were successfully inherited in consecutive generations. Six mutated lines viz., KS2-12-1-3, KS2-27-4-1, KS2-45-6-1, KS2-70-1-2, KS2-75-1-11, KS2-144-1-2 were selected, which displayed a drastically reduced number of blast lesions and were at par with the agronomic traits when compared with the wild types. Similarly, Zhou et al. [377] attempted to mutate three broad-spectrum blast-resistant genes, i.e., Bsr-d1, Pi21, and ERF922, using CRISPR-based technology in Longke638S (LK638S) rice cultivar which is a TGMS line. They observed that single and triple mutants had improved resistance against blast. Furthermore, the improved varieties also had similar kinds of desirable superior agronomic characteristics. In another experiment, CRISPR Cas9 was used to target S genes, Pi21 and Bsr-d1 in rice which was responsible for the susceptible reaction with rice blast pathogen *Magnoporthe oryzae* [378]. Due to gene editing, resistance was conferred by two methods, firstly by generating a loss of function mutants and secondly by generating knock of mutants [378]. Similarly, the OsSEC3A gene mutated using targeted mutagenesis, which enhanced resistance to rice blast disease [379]. Many scientists have also utilized the CRISPR Cas9-based gene editing approach to develop resistance in rice against the blast disease [64,379,380], which is presented in Table 6.

#### 7.5.2. Transcription Activator-like Effector Nucleases (TALENs)

TALENs induce site-specific double-strand breaks (DSBs) in DNA [381]. These breaks can be amended by homologous recombination (HR) or non-homologous end-joining (NHEJ). The bacteria of Xanthomonas species secrete proteins known as TALEs (Transcription activator-like effectors) which display pathogenic activity in crops such as rice, tomato, etc. [382]. Further research revealed that these proteins could recognize and bind to specific sequences in DNA [383]. This was followed by the discovery of code which recognized specific regions of DNA [384]. The one monomer nucleotide property of TALE fascinated many researchers to work with it. This technique was used to combat filamentous fungus such as *Magnoporthe oryzae*, and an HR detection-based PtFg TALEN plasmid was constructed. This highly efficient nuclease can improve the efficiency of HR-mediated gene editing up to 100% and can prove to be very important for developing resistant varieties against various strains of *Magnoporthe oryzae* [385].

#### 7.5.3. Meganucleases (MNs)

These are also known as homing endonucleases, which are divided into five types based on their sequence and structure motifs [386], from which the LAGLIDADG family proteins have the capacity to act as endonucleases that can bind to specific sites on the DNA [387]. They can recognize exon- or intron-free regions and bind with longer DNA sequences (14 to 40 bp), and induce double-strand breaks (DSBs) in DNA [388]. MNs are not commonly used because naturally occurring endonucleases are very limited and can recognize only a few sites on the genome. Artificial MNs require a huge input of time and money which requires a lot of sustained effort and is practically not feasible. Despite having limited usage, this technique can be kept as an alternative to generate novel genetic changes to develop resistance against various strains of *Magnoporthe oryzae*.

#### 7.5.4. Zinc Finger Nucleases (ZFNs)

ZFNs are counterfeit for natural endonucleases known as Fokl and are derived from Flavobacterium okeanokoites [389]. They consist of two terminal ends, the N terminal, which binds with DNA, and the C terminal, which has cleavage activity. Each ZFN is capable of identifying a sequence of 3–4 nucleotides. Different ZFNs can be combined to recognize a longer sequence and can be used to induce DSBs in DNA for genome editing [390]. Later, using NHEJ or HR repairs, the required nucleotide sequence or gene can be inserted to achieve targeted results. ZFNs can be introduced into hosts using viral or non-viral vectors and have the capacity to incorporate themselves into any genome, including that of mitochondria [391]. ZFNs have been successfully used to induce mutations or edit genomes in various crops. An experiment was conducted on rice to identify safe sites to introduce new genes using three different ZFN constructs (pZFN1, pZFN2, pZFN3), which were delivered using Agrobacterium and β-glucuronidase (GUS) as a reporter gene. Gene expression was measured using TAIL PCR which identified 28 presumed safe sites which can be used to harbor genes of interest for improving resistance against diseases such as rice blasts [392].

## 8. Problems Associated with Breeding for Resistance to Rice Blast 

Boosting the yield potential and productivity of rice is the primary objective for the plant breeders; however, in complementation, resistance breeding for biotic and abiotic stress has been considered a major issue due to changing climatic conditions [43]. Conventional breeding methods for developing blast-resistant rice varieties are tedious and time-consuming as it takes about 8–10 years to develop one resistant variety. In parallel, *Magnoporthe oryzae* is mutating continuously and evolving new races against the resistant genes available in the existing cultivars within 2–3 years. In such cases, the resistant cultivar becomes susceptible, leading to discouragement to the breeders as their long efforts have been destroyed quickly. Moreover, low-yielding cultivars that have blast resistance have not been preferred by the farmers and farming community; therefore, breeding for high-yielding rice varieties with multiple durable R-genes for blast resistance is the need of the hour. Advancement in genomic approaches has enabled the breeders to accurately introgress or pyramid multiple R-genes in the high-yielding desirable genotypes within a short period with the help of molecular markers and genomic selection [126,317]. However, linkage drag is the major issue in the case of gene introgression and pyramiding. A number of undesirable changes due to linkage drag in the recipient genotype led to their poor yield performance, and, fortunately, that cannot be compromised by the farmers [19,20,347]. In the current era of genome editing, several scientists have developed blast-resistant and high-yielding rice varieties using genetic engineering and cisgenesis [12,372,378]. However, they have not been accepted worldwide due to the imposition of regulations on genetic modification in many rice-producing and rice-importing countries. Nonetheless, no genetically modified disease-resistant rice is currently commercially available for production worldwide [43]. While these techniques have been proven successful in experimental settings, they have not been made commercially available in rice due to regulatory protocols put in place by many rice-importing countries.

## 9. Conclusions and Future Outlooks

**Figure 5:**
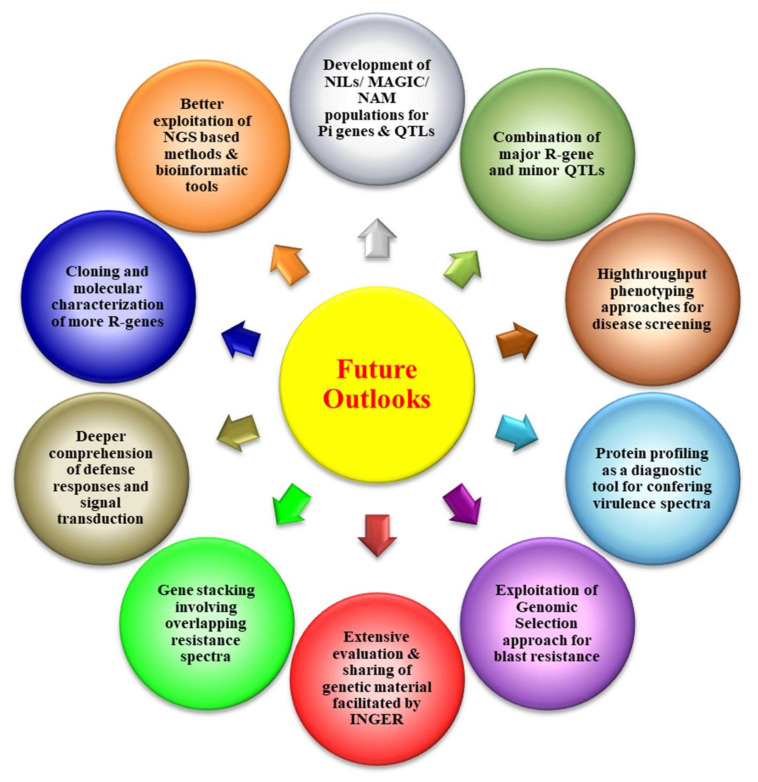
Future perspectives and way forward for developing blast resistance in rice.

It is well understood that combining the multiple race-specific R genes in elite cultivars is the most effective and appropriate strategy for developing broad-spectrum and durable resistance to blast disease. However, multiple R-genes in cultivars may promote the evolution of several new races of the pathogen, and even super races could arise, which may cause severe blast epidemics by overcoming the multiple major R-genes. Therefore, it is crucial to rationally utilize the race-specific R-genes in breeding programs to sustain the blast resistance of rice cultivars which is still poorly understood. This opens many avenues for researchers to explore blast resistance in rice. A diagrammatic representation of the possible aspects for further improvement in blast resistance is represented in Figure 5. The major obstacle in managing rice blasts is the durability of the genetic resistance. Thus, the right combination of the major R gene and minor QTLs is necessary to confer broad-spectrum and durable resistance with the help of conventional integrated breeding, advanced genomic approaches, and genome editing tools. 

Interestingly, of the 36 molecularly characterized and cloned R genes, several broad-spectrum genes are available for further use in breeding programs. This indicates that along with the mining of novel genes, their cloning and characterization are also important. Furthermore, high-quality Pi genes, which are less prone to evasion by the pathogen and impose fitness penalties in mutations of the pathogen, are expected to be effective and durable. Work on these genes could be accelerated. As neck blast is more damaging to yield and grain quality, screening of neck blast resistance should be incorporated in key evaluation sites. A rice variety, ‘Mahamaya’ of Chhattisgarh, India, is very popular for flaked rice. However, it is susceptible to neck blasts; therefore, there is a need to incorporate resistant genes against the neck blast. Until now, only a few genes have been identified showing the true resistance against the panicle and leaf blast; therefore, mining the R-genes/QTLs imparting equal effectiveness against panicle blast and leaf blast diseases would enhance the resources for the breeders. Moreover, disease screening protocols can be improvised through high throughput phenotyping approaches which could help in better monitoring and management of the disease. With the available genome sequence of the blast fungus, comprehensive profiling of secreted proteins of the fungus is now possible. These proteins may confer effector functions and can act as a diagnostic tool for determining the virulence/avirulence spectrum of a given pathogen population.

Furthermore, the ability to evaluate genetic materials at multiple sites is essential to assess the spectrum of resistance of breeding lines and gain a glimpse of the potential durability. Performance records are organized by the International Network Genetic Evaluation of Rice (INGER) and are used as an indicator of the durability of resistance in varieties. This will require extensive sharing of genetic materials between countries, perhaps through the facilitation of INGER. Gene stacking involving major R genes with overlapping resistance spectra involving superior alleles would confer durable resistance. Integrating advanced breeding methods, modern molecular approaches along with next-generation sequencing (NGS) based methods, and bioinformatic tools will effectively direct in achieving rice blast resistance. High-throughput whole genome sequence (WGS) or target gene sequencing in the elite rice cultivars or core resistant germplasm will provide useful technological means for breeding selection. 

In addition, genomic selection should be actively utilized in breeding for blast disease resistance as it exploits the genomic-estimated breeding values of individuals obtained from genome-wide markers to choose candidates for the next breeding cycle. The availability of a standard phenotyping approach and genome-wide high-throughput, cost-effective and flexible markers, especially with the emergence of NGS techniques, has enabled the plant breeders to exploit genomic selection (GS) for crop improvement. The NGS-based genotyping approaches, such as genotyping by sequencing, have significantly improved the prediction level of genomic-estimated breeding values in cereals and other crop species as compared to the other established marker platform and helped in deploying the GS in breeding programs. Furthermore, GS is the most suited approach for breeding for quantitative resistance conferred by minor effect genes, or a combination of minor and major genes tends to produce a more durable resistance in breeding lines (BLs) because it relies on multi-resistant alleles. In addition to increasing the accuracy of selection, GS is expected to reduce rates of inbreeding because the increased accuracy of Mendelian sampling terms in GS allows for the identification and selection of elite breeding candidates from more families, with lower co-selection of sibs. Improved statistical models that leverage genomic information to increase prediction accuracies are critical for the effectiveness of GS-enabled breeding programs.

More studies towards a deeper comprehension of defense responses and signal transduction leading to defense responses are required. NILs (Near Isogenic Lines), Multi-parent advanced generation inter-cross (MAGIC) populations, and Nested Association Mapping (NAM) are useful genetic resources for genetic analysis and, eventually, gene cloning. More NILs, MAGIC, and NAM populations for diverse and durable Pi-genes and QTLs could be developed primarily for genetic analysis and for monitoring pathogen populations. Marker-aided foreground and background selection can be used to accelerate NIL development. More expertise could be involved in performing recently developed transgenic and genome editing tools for its better exploitation to develop blast resistance in rice through specific genetic modifications. Broad spectrum resistance may arise due to the mutations in susceptibility genes in plants. Such loss of function mutations in susceptibility genes indirectly imparts disease resistance without yield penalty. Future studies must orient towards the mining of such S genes in rice in order to utilize them through genome modification techniques for developing blast-resistant varieties.

## Figures and Tables

**Table 1:** List of blast-resistant rice mutant varieties developed across the World.

S. No.	Name of Mutant Variety *	Registration Year in MVD	Country	Mutagen Used and Dose	Character Improvement Details
1	Xiongyue 613	1965	China	Gamma rays (200 Gy)	Moderate resistance to blast, higher yield and good quality
2	Fulianai	1966	China	Gamma rays (200 Gy).	Short culm, resistance to blast, early maturity and high yield
3	Aifu 9	1966	China	Gamma rays (350 Gy)	Short culm, resistance to blast and higher yield
4	Liaofeng 5	1969	China	Gamma rays (250 Gy)	Early maturity, short culm and resistance to blast
5	Fuxuan 3	1970	China	Gamma rays (300 Gy)	Good tillering and resistance to blast
6	Fushe 94	1971	China	Neutrons	Early maturity, good tillering and resistance to blast
7	Nucleoryza	1972	Hungary	Fast neutrons (25 Krad)	Early maturity, maintained blast resistance and improved yield
8	Fuxuan 124	1972	China	Gamma rays (350 Gy)	Resistance to blast and intermediate maturity
9	Yifunuo 1	1973	China	Gamma rays (100 Gy)	Resistance to blast, long panicles and higher grain number
10	Fulgente	1973	Italy	X-rays (250 Gy)	Blast resistance and high productivity
11	Fushe 410	1974	China	Gamma rays (350 Gy)	Intermediate resistance to blast
12	Wangeng 257	1975	China	Gamma rays (300 Gy)	Tolerance to fertilizers, resistance to blast and higher yield
13	Nongshi 4	1975	China	Neutrons	Early maturity, resistance to low temperature, resistance to blast and xantomonas oryzae
14	Xiangfudao	1976	China	Gamma rays (300 Gy)	Resistance to low temperature, resistance to blast and xantomonas
15	RD 6	1977	Thailand	Gamma rays (200 Gy)	Glutinous endosperm and improved resistance to blast
16	Guifu 3	1977	China	Gamma rays (300 Gy)	Early maturity, resistance to low temperatures and resistance to blast
17	7404	1977	China	Gamma rays (350 Gy)	Short culm, higher yield, resistance to bacterial blight and blast
18	Wanfu 33	1978	China	Gamma rays (300 Gy)	Early maturity, resistance to low temperatures and resistance to blast
19	Zhuqin 40	1978	China	Gamma rays (300 Gy)	Resistance to blast and suitable for mountain areas
20	Juangyebai	1978	China	Neutrons	Good tillering and resistance to blast
21	Fuzhu	1979	China	Gamma rays (350 Gy)	Early maturity, resistance to low temperatures, resistance to blast
22	Jagannath (BSS-873)	1979	India	X-r (300 Gy)	Wide adaptability, semi dwarf, resistance to blast and sheath blast
23	Mutashali	1980	Hungary	Fast neutrons (20 Gy)	Resistance to blast and shattering of grains and high yield
24	Atomita 1	1982	Indonesia	Gamma rays (200 Gy)	Early maturity, resistance to bph (biotype 1), green leaf hopper and blast
25	CNM 31	1982	India	X-rays (300 Gy)	Early maturity, semidwarf, higher yield, resistance blast
26	Atomita 2	1983	Indonesia	Gamma rays (200 Gy)	Tolerance to salt, early maturity, resistance to brown plant hopper (biotype 1), higher protein content and resistance to blast
27	Danau atas	1988	Indonesia	Gamma rays (400 Gy)	High yield, resistance to blast, drought and low ph
28	Xiangjing 832	1989	China	X-rays	Short straw, high resistance to blast and bacterial blight, high yield
29	CRM 49	1989	India	0.001 m Sodium azide (Nan3)	Resistance to blast disease
30	Quannuo 101	1990	China	Gamma rays (200 Gy)	High grain yield, wine making rice, moderately resistant to blast and bacterial leaf blight
31	Jinfu 1	1990	China	Gamma rays (300 Gy)	Early maturity (7 days earlier) and resistance to blast
32	Ejingnuo 6	1991	China	Gamma rays (350 Gy)	Resistance to blast and blight, good grain quality and higher grain yield
33	Xiushui 04	1991	China	Physical mutagen	Resistance to blast and bacterial blight, high yield, good grain quality and altered maturity
34	Zhenuo 2	1993	China	Gamma rays (300 Gy)	High grain yield, good cooking quality, resistance to rice blast and bacterial blight
35	Fuyou 63	1993	China	Physical mutagen	High grain yield, altered maturity, blast resistance, 21.98% amylose content
36	Zhefu 762	1993	China	Physical mutagen	High grain yield, high resistance to blast and bacterial blight
37	Zhefu 7	1994	China	Gamma rays (300 Gy)	Early maturity, resistance to low temperature, resistance to blast and sheath blight
38	II You 838	1995	China	Physical mutagen.	High grain yield, plant height (120 cm), resistance to leaf blast and panicle blast, amylose content (22.8%)
39	Shengxianggeng No. 4	1996	China	Gamma rays (180 Gy)	Short stem, high yield and good quality, high resistance to rice blast
40	Camago-8	1996	Costa Rica	Gamma rays (250 Gy)	Resistance to blast and resistance to viruses
41	CRM 53	1997	India	0.66% EMS	Resistance to blast disease
42	VND 95-19	1999	Viet Nam	Gamma rays (200 Gy)	Strong tolerance to acid sulphate soil, high yield (5–10 t/ha), resistance to brown plant hopper and blast disease
43	VND 95-20	1999	Viet Nam	Gamma rays (200 Gy)	Short duration (90–95 days), wide adaptation, intermediate resistant to brown plant hopper, blast disease
44	CNM 25	1999	India	X-rays (300 Gy)	Early maturity, increased tillering, higher yield, moderately resistant to blast
45	CNM 6 (Lakshmi)	1999	India	X-rays (300 Gy)	Early maturity (15–23 days), resistance to drought, dwarf (85 cm), and moderately resistant to blast
46	Yueyou 938	2000	China	Gamma rays	High yield, semi dwarf plant height, resistance to bacterial blight and blast
47	Radhi	2000	India	Gamma rays (250 Gy)	Tolerance to blast and bph, good yield and early maturity (120 days)
48	IACuba 28	2001	Cuba	Fast neutrons (20 Gy)	Large grain size, high yield, resistance to blast
49	CRM 51	2003	India	0.001 m Sodium azide (Nan3)	Resistance to blast disease
50	Woncheongbyeo	2003	Korea	Gamma rays (300 Gy)	Short stature, resistance to blast and early maturity
51	Zhongzao 21	2003	China	Na	Medium maturity, tillering ability, good grain quality, blast resistance
52	Yangfujing4901	2004	China	Gamma rays	Strong resistance to blast, bacterial leaf blight
53	Pooya	2004	Iran	Gamma rays (150 Gy)	Resistance to lodging and blast and higher yield
54	Tabesh	2004	Iran	Gamma rays (150 Gy)	Resistance to lodging, short culm, tolerance to blast and higher yield
55	VND99-3	2004	Viet Nam	Gamma rays (200 Gy)	Short duration, high yield, resistant to brown plant hopper, blast disease,
56	Yangfuxian 9850	2004	China	Gamma rays (300 Gy)	High yield, medium maturity, excellent eating quality, resistance to blast,
57	Chiyou S162	2005	China	Gamma rays (300 Gy)	Moderate plant height, moderate tillering ability, resistance to blast and bacterial blight
58	Zhe 101	2005	China	Treatment of seeds in aerospace	Late maturity, high yield, resistance to blast and bacterial blight
59	Pusa-NR-546	2006	India	Gamma Rays (300 Gy)	Grain quality, semi dwarf (100 cm), super fine grain, tolerance to brown spot and leaf blast,
60	Hangtian 36	2006	China	Treatment of seeds in aerospace	Early maturity, high grain quality and blast resistance
61	Huahang-simiao	2006	China	Treatment of seeds in aerospace	Resistance to blast and good quality
62	Minami-yutaka	2007	Japan	Gamma rays (200 Gy)	Late maturity, resistance to lodging, leaf blast and panicle blast
63	Jahesh	2008	Iran	0.001% EMS	Short stature, early maturity, high yield and tolerant to stem borer and blast disease
64	Partou	2008	Iran	Gamma rays (350 Gy)	Short stature, early maturity, high yield, tolerant to stem borer and blast disease
65	Guangyinruanzhan	2008	China	Physical mutagen	High yield, resistance to blast and bacterial leaf blight
66	Liangyouhang 2	2008	China	Treatment of seeds in aerospace	High yield, resistance to blast and bacterial blight and good grain quality
67	Neiyouhang 148	2008	China	Treatment of seeds in aerospace	High yield, blast resistance and late maturity
68	Zhejing 41	2009	China	Physical mutagen	Medium maturity, high yield, the resistance to blast and bacterial leaf blight
69	SCS118 Marques	2013	Brazil	Gamma rays (300 Gy)	Moderate resistance to blast, high yield potential,
70	NMR 151	2015	Malaysia	Gamma rays (300 Gy)	Minimal water requirement, tolerant to blast disease, and high yield
71	Roshan	2019	Iran	Gamma rays (250 Gy)	Short stature, early maturity, tolerant to stem borer and blast disease
72	Vikram-TCR	2021	India	Gamma rays (300 Gy)	Semidwarf, Mid-early Maturity, High Yielding and Resistant to Blast Disease

* Source: All the information have been collected from FAO/IAEA MVD (2022). https://nucleus.iaea.org/sites/mvd/SitePages/Home.aspx (accessed on 25 June 2022).

**Table 5:** Deployment of transgenic approaches for developing blast resistance in rice.

S. No.	Donor Organism	Transferred Gene	Gene Function	Technique Used	Host Organism	Reference
1	Wild rice	MoHrip1 and MoHrip2	Imparts resistance against blast and improvement in agronomic traits	*Agrobacterium tumefaciens* mediated transfer	*Oryza sativa* L.	Wang et al. [29]
2	Wild rice	*Cht-2 and Cht-3*	Formation of chitin	*Agrobacterium tumefaciens* mediated transfer	*Oryza sativa* L. *japonica* (Nipponbare and Koshihikari varieties)	Nishizawa et al. [363]
3	Wild rice	Gns1	Hydrolysesglucosidic bonds in cell walls	Introduced through vectors by incooperating CaMV35S as its promoter	*Oryza sativa* L.	Nishizawa et al. [365]
4	Wild rice	Pi54of	Confers resistance against blast	Transferred using pET29a vector	Two susceptible rice lines IET16310 (*indica*) and TP309 (*japonica*)	Devanna et al. [28]
5	Wild rice	*Pi-d2*	Confers resistance against blast	Vector mediated transformation	*Oryza sativa* L.	Chen et al. [369]
*6*	*Oryza rhizomatis*	*Pi54rh*	Confers resistance against blast	-	*-*	Das et al. [287]
7	Giant silk moth *Hyalophora cecropia*	ER-CecA	Produce *scecropin* A peptides in paddy which are antimicrobial protein	Vector mediated transformation	*Oryza sativa* L.	Coca et al. [366]
8	Artificially made	Thanatin	Antimicrobial protein	Vector mediated transformation	*Oryza sativa* L.	Imamura et al. [368]
9	Maize	C2	Flavanoid production	pUOH series plasmids	*Oryza sativa* L.	Gandikota et al. [364]
10			Antifreeze glycopeptide gene	*Agrobacterium tumefaciens* mediated transfer	*Oryza sativa* L.	Zhang et al. [367]
11	Wild rice	RC24	An alfalfa glucanase gene,	Biolistics	*Oryza sativa L. indica* var. *Qisiruanzhan*,	Feng et al. [371]
12	Wild rice	Beta-Glu	Beta glucanase	Biolistics	*Oryza sativa L. indica* var. *Qisiruanzhan*,	Feng et al. [371]
13	Barley	B-RIP	Ribosome-inactivating protein	Biolistics	*Hardeum vulgare*	Feng et al. [371]
14	Wild rice	*Pi21*	Confers resistance against blast	*Ac/Ds* transposon vectors	*Oryza sativa* L.	Li et al. [30]
15	Rice	ACS2	1-aminocyclopropane-1-carboxylic acid synthase	Vector mediated transformation	*Oryza sativa* L.	Helliwell et al. [370]
16	Rice	Pib, Pi25 and Pi54	Confers resistance against blast	Vector mediated transformation	*indica* variety Kasalath and the *japonica* variety Zhenghan 10	Peng et al. [31]

**Table 6:** Potential use of CRISPR Cas9 for resistance against *Magnaporthe oryzae*.

S. No.	Plant Species	Target Gene	Gene Function	Strategy	Reference
*1*	*Oryza sativa* L. *japonica*	SEC3A	Subunit of the exocyst complex	Protoplast transformation with Cas9/gRNA expression binary vectors	Ma et al. [379]
*2*	*Oryza sativa* L. *japonica*	ERF922	Transcription factor implicated in multiple stress responses	*Agrobacterium*-mediated transformation of embryogenic calli with Cas9/gRNA expression binary vectors	Wang et al. [97]
*3*	*Oryza sativa* L.	*ALB1* (MGG_07219).	Polyketide synthase	Poisons the fungus by converting it into albino colour.	Foster et al. [64]
*4*	*Oryza sativa* L.	*RSY1(MGG_05059)*	Scytalone dehydratase enzyme	Poisons the fungus by converting it into orange-red (rosy) fungal colonies	Foster et al. [64]
*5*	*Oryza sativa* L.	*OsERF922*	ABA accumulation	Number of blast lesions formed were less, improving blast resistance	Wang et al. [97]
*6*	*Oryza sativa* L.	*OsMPK5*	Responsible for pathogen infection	Improves resistance against blast diseases	Xie and Yang [380]
*7*	*Oryza sativa* L.	*OsERF922*	Responsible for pathogen infection	Improves resistance against blast diseases	Wang et al. 2016 [97]
*8*	*Oryza sativa* L.	*ALB1, RSY1*	Aids in growth of pathogen	Ribonucleoprotein (RNP) based CRISPR induced, marker free resistant plants against blast	Xie and Yang [380]
*9*	*Oryza sativa* L.	*OsSEC3A*	Interacts with SNAP25-type t-SNARE protein OsSNAP32 which is responsible for blast resistance	Induces plant defense responses for *Magnoporthe oryzae.*	Ma et al. [379]
10	*Oryza sativa* Longke638S (LK638S)	SA and JA pathway associated genes	Improves plant immunity	Increases resistance against blast.	Zhou et al. [377]
11	*Oryza sativa* L.	S genes, Pi21 and Bsr-d1	Responsible for susceptible reaction in rice for blast.	Increases resistance by knocking out S gene or by causing mutation	Tao et al. [378]

## Data Availability

Not applicable.

## Supplementary Materials

The following supporting information can be downloaded at: https://www.mdpi.com/article/10.3390/plants11182386/s1, Table S1: List of blast disease resistant rice varieties developed through crossing with mutant varieties.
