# Supplementary material for: Advancement in the Breeding, Biotechnological and Genomic Tools towards Development of Durable Genetic Resistance against the Rice Blast Disease
Source: Plants (Basel). 2022 Sep 13;11(18):2386. doi: 10.3390/plants11182386 (PMC9504543; doi:10.3390/plants11182386)
Supplement: Supplementary file 1 [file plants-11-02386-s001.zip › plants-1862219-supplementary.pdf]

Table S1. List of blast disease resistant rice varieties developed through crossing with mutant varieties.

S. No.	Name of Mutant Variety	Registration Year in MVD	Country	Mutagen Used & Dose	Character Improvement Details
1	Gayatri (IET-8020)	1975	India	hybridization with mutant variety Jagannath	Semi-dwarfness (100 cm), short grains, high yield, resistant to BLB, moderately resistant to blast and GM (gall Midge)
2	Nadahikari	1977	Japan	hybridization with one mutant Hyokeisake 18 obtained by irradiation with gamma rays.	Short culm, high grain yield and resistance to leaf blast
3	Shivam	1978	India	hybridization with mutant CR 314-5-10	semi-dwarfness, (95–110 cm), grains (short bold), highly resistance for blast, Gall Midge biotype 4, Sheath blight and leaf folder, yield (45-50 Q/ha) and maturity duration (115-125 days)
4	Katsurawase	1978	Japan	hybridization with one mutant induced by irradiation with gamma rays.	Short and stiff culm, early maturity and resistance to blast
5	Sachiminori	1978	Japan	hybridization with one mutant R4-B induced by irradiation with gamma rays.	Stiff culm and resistance to blast
6	Yawarakomachi	1980	Nigeria	hybridization with mutant line derived from variety Kinmaze treated with chemical mutagen NMU.	Low amylose content and good quality, It has a true resistant gene to the blast, Pia and the field resistance is slightly weak before heading and becomes moderate after heading
7	Kefuhong 2	1981	China	hybridization with mutant IR8	Early maturity and resistance to blast
8	Xiushui 48	1981	China	hybridization with mutant Funong 709	Resistance to blast, tolerance to low temperature, tolerance to day length and high yield
9	Xianghu 24	1983	China	hybridization with mutant Funong 709 [(Funong 709 x Jingyin 154) x Funong 709	Resistance to blast and blight, tolerance to day length and glutinous grain type
10	PNR-519	1983	India	gamma rays	Semi-dwarfness (100 cm), brown long slender grains, resistance to blast, Sheath blight, gall midge, leaf folder, yield (55 q/ha), maturity (85-115 days)
11	Pusa-NR-381	1985	India	hybridization with mutant Tainan-3 induced by	Resistance to blast

				irradiation with gamma rays	
12	Minnuo 706	1988	China	gamma rays (250 Gy)	Good tillering, higher yield, glutinous, resistance to blast, good quality
13	Daisenminori	1988	Japan	hybridization with mutant Tori Kei 4 (dwarf mutant of Norin 8 induced by irradiation with gamma rays).	Resistance to lodging, high yield, good cooking quality and resistance to blast
14	Michinoku-wase	1988	Japan	hybridization with mutants descendant of mutant variety Reimei obtained by irradiation of seeds with gamma rays (200 Gy).	Early maturity, resistance to leaf blast, high yield and good quality
15	Keshari	1989	India	X-rays	Short stem, moderately resistant to GLH (Green Leaf hopper), blast and BLB, high yield and early maturity
16	Hae-nuki	1993	Japan	hybridization with mutant line derived from the mutant variety Reimei induced by irradiation of seeds with gamma rays (200 Gy).	Late maturity, high yield, good quality, resistance to lodging and resistance to blast disease
17	Fukuhibiki	1995	Japan	hybridization with mutant variety Dontokoi obtained by irradiation with gamma rays.	High yielding ability, excellent plant type, high tolerance to lodging and suitable for sake brewing
18	Fu 8970	1995	China	hybridization with two mutants Fu 85151 and Fu 8638 obtained by physical irradiation	High grain yield, improved seed production traits, big spike, higher grain number, higher grain weight, moderate maturity, compact plant type, resistance to blast and bacterial leaf disease, resistance to white-backed planthopper
19	II You 802	1996	China	hybridization with one mutant Chuanhui 802 obtained by irradiation with gamma rays	High grain yield, growth period (150 days), late maturity, compact plant type, strong tillering, more grains per panicle, grain weight (28 g), blast resistance
20	Fuyou 130	1997	China	hybridization with Fu 76A x 130	Superior yield (12.5%), average height (116.1 cm), tolerance to low temperatures, strong tillering ability, blast resistance
21	Hashiri-aji	1997	Japan	by hybridization with mutant variety Niigatawase induced by irradiation of seeds with	Early maturity, resistance to lodging, resistance to rice blast and good quality

				gamma rays (200 Gy)	
22	Lafitte	1997	United States	hybridization with mutant Mercury (cross Mercury/Mercury//Koshihikari) obtained by irradiation of seeds with gamma rays.	Semi-dwarfness, early maturity and resistance to blast
23	Wandao 51	1997	China	hybridization with mutant	High grain yield, good quality, multiple resistance, bacterial blight resistance, resistance to blast and insects
24	Fuyou 838	1997	China	hybridization with two mutants Fu74A and Fuhui 838	Superior yield (12.5%), average yield of 7620 kg per hectare, average height (115 cm), thick stem, compact plant growth, light green leaves, average of 150 grains per panicle, blast resistance
25	Soft 158	1997	Japan	hybridization with mutant variety Kinuhikari induced by gamma rays and mutant Ken-kei 2078 obtained by treatment with chemical mutagen EMS	Low amylose content, true resistance gene to rice blast, Pi-a and moderate field resistance to blast
26	Asamurasaki	1998	Japan	hybridization with mutant variety Fukuhibiki induced by irradiation of seeds with gamma rays (200 Gy)	has true resistance gene of Pi-a and high field resistance to leaf blast, and good cooking quality
27	Fuyou 802	1998	China	hybridization with one mutant Fu74A.	High grain yield (10.9%), , altered maturity, growth period (146 days), strong tillering ability, average height (109.4 cm), ear length (24 cm), blast resistance, medium-grain quality
28	Savitri	1999	India	Crossing with one mutant (Jagannath)	Day length sensitivity, late maturity, wide adaptability, semi-dwarf (110-120 cm), short grains, good milling recovery, tolerant to blast and sheath blight and high yield
29	Xieyou 371	1999	China	gamma rays (10 Gy)	High grain yield (10.1%), improved plant structure, growth period (126 days), resistance to blast, bacterial leaf blight and bacterial leaf streak, resistance to white-backed planthopper and brown planthopper
30	Tsukushi-wase	1999	Japan	hybridization with mutant variety Kinuhikari obtained by	High yield, short culm, resistance to lodging and

				irradiation with gamma rays	early maturity, It has the blast resistance gene Pi-i.
31	Haiminori	2000	Japan	hybridization with mutant line derived from Kinmaze treated with chemical mutagen NMU.	medium to late maturity and resistance to blast disease
32	Okini-iri	2000	Japan	hybridization with mutant line derived from the mutant variety Reimei induced by irradiation of seeds with gamma rays (200 Gy).	Superior eating quality and high field resistance to blast
33	Ideyu-mochi	2000	Japan	hybridization with mutant line derived from the mutant variety Reimei induced by gamma rays.	Early-maturity, high-yielding variety with excellent processing quality of rice cakes, It has major resistant gene to rice blast, and has slightly-strong field resistance to leaf blast and has moderate field resistance to panicle blast.
34	Yawarakomachi	2000	Japan	hybridization with mutant line derived from variety Kinmaze treated with chemical mutagen NMU.	Low amylose content and good quality, number of panicles is slightly greater. It has a true resistant gene to the blast
35	Aki-no-sei	2000	Japan	hybridization with mutant variety Miyama-nishiki induced by irradiation with gamma rays (300 Gy	High percentage of white core rice and suitable for sake-brewing, resistance to leaf and panicle blast
36	Asahi-no-yume	2000	Japan	hybridization with mutant variety Aichinokaori and mutant line derived from the mutant variety Reimei, both induced by irradiation of seeds with gamma rays (200 Gy	Early maturity, high yield, highly resistant to rice stripe disease and panicle blast
37	Oyama-nishiki	2001	Japan	hybridization with mutant line derived from the mutant variety Miyama-nishiki induced by gamma rays (300 Gy)	High percentage of white core rice, suitable for sake brewing and show blast resistance
38	Hanabusa	2002	Japan	hybridization with mutant line Douhoku No. 53, a sister line of mutant variety Aya obtained by irradiation with gamma rays	Low amylose content (average 14%), boiled rice has high stickiness, good eating quality, medium maturity, more resistant to cool weather and blast disease
39	Koimusubi	2002	Japan	hybridization with mutant of mutant variety Mine-asahi obtained by irradiation of seeds with gamma rays (200 Gy)	Excellent cultivation characteristics, highly cold tolerance, blast resistance and excelent eating quality
40	Koshihikari Niigata BL No. 4	2002	Japan	by hybridization with mutant derived from mutant variety Reimei obtained by irradiation	Resistance to blast

				of seeds with gamma rays (200 Gy)	
41	Yume-akari	2002	Japan	hybridization with mutant line derived from the mutant variety Reimei induced by irradiation of seeds with gamma rays (200 Gy)	Medium maturity, high eating quality and tolerance to low temperatures and show resistance to blast
42	Chiho-no-kaori	2002	Japan	hybridization with mutant variety Hagi-no-kaori induced by irradiation of seeds with gamma rays (200 Gy)	High yield, good quality, strong resistance to leaf blast and panicle blast
43	Daichi-no-kaze	2002	Japan	hybridization with mutant variety Matsuri-bare induced by irradiation of seeds with gamma rays (200 Gy)	Medium maturity, high yield, good eating quality, resistance to rice stripe disease and panicle blast, moderately resistant to bacterial leaf blight
44	Benigoromo	2002	Japan	hybridization with mutant variety Fukuhibiki obtained by irradiation of seeds with gamma rays (200 Gy).	Early maturity, short stem, high yield, large red grains, has a true resistance gene Pia to blast [Magnaporthegrisea], and the field resistance to leaf and panicle blasts are slightly weak and moderate, respectively.
45	Misato-nishiki	2002	Japan	hybridization with mutant variety Miyama-nishiki obtained by irradiation with gamma rays (300 Gy)	Early maturity and high quality, blast disease resistance gene is estimated to be Pii type.
46	Yume-sayaka	2003	Japan	hybridization with mutant line derived from the mutant variety Reimei induced by irradiation of seeds with gamma rays (200 Gy).	Very early maturity, nonglutinous rice variety, resistance to leaf blast and good quality
47	Gin-otome	2003	Japan	hybridization with mutant induced by gamma rays	Short culm, suitable for sake brewing
48	Oborozuki	2003	Japan	It was developed by hybridization with mutant from in vitro culture	Low amylose content, good eating quality and high tolerance to low temperatures, field resistance to leaf blast is moderate to low
49	Oku-no-murasaki	2003	Japan	hybridization with mutant line derived from the mutant variety Reimei induced by irradiation of seeds with gamma rays (200 Gy).	Nonglutinous purple grains, lodging resistance moderate eating quality. It has a true resistance gene Pib to blast.
50	Aichi-no-kaori SBL	2003	Japan	hybridization with mutant variety Aichinokaori induced by irradiation of seeds with gamma rays (200 Gy).	High resistance to rice stripe disease and panicle blast and moderately resistance to bacterial leaf blight

51	Mine-hibiki	2003	Japan	hybridization with one mutant Mineasahi obtained by irradiation with gamma rays (200 Gy).	Extremely early maturity, lodging resistance, superior eating quality, high resistance to leaf and panicle blast
52	Yume-no-kaori	2003	Japan	cross between "Hattannishiki 1" and "YamagataSake 49 (cultivar Dewasansan obtained by irradiation with 300 Gy gamma rays	Good eating quality, suitable for sake brewing, good lodging resistance and resistance to blast
53	Yume-shizuku	2003	Japan	by hybridization with mutant variety Kinuhikari induced by irradiation with gamma rays	Early maturity, resistance to blast and good eating quality
54	Chiyo-no-mochi	2003	Japan	hybridization with mutant line derived from the mutant variety Reimei and mutant variety Yama-uta induced by irradiation of seeds with gamma rays (200 Gy	Medium maturity, cool weather tolerance and high yielding ability
55	Fusa-no-mai	2004	Japan	hybridization with mutant varieties Reimei and Mine-asahi induced by irradiation of seeds with gamma rays (200 Gy)	high cold resistance, high resistance to the panicle blast
56	Fukumirai	2004	Japan	hybridization with mutant variety Mine-asahi induced by irradiation of seeds with gamma rays (200 Gy)	Non glutinous paddy rice, high yield and good quality
57	OM2717	2004	Viet Nam	hybridization with mutantn variety TNDB 100 obtained by irradiation of seeds with gamma rays (50 Gy).	Short duration (90-95 days), high yield (4-7 T/ha), good quality (long grain, amylose content: 20-24%),wide adaptation, intermediate resistant to brown plant hopper and blast disease
58	OM2718	2004	Viet Nam	gamma rays (50 Gy)	Short duration (90-95 days), high yield (5-8 t/ha), good quality, wide adaptation, intermediate resistant to brown plant hopper and blast disease
59	Hyo-kei-sake No. 65 (Toji-no-hana)	2004	Japan	hybridization with mutant variety Hyogo-Kitanishiki obtained by irradiation with gamma rays	Extremly early maturity, high resistance lodging, resistance to rice blast disease, high quality and high yield
60	Mie-no-yume	2004	Japan	hybridization with mutant variety Matsuri-bare obtained by irradiation of seeds with gamma rays (200 Gy)	Medium maturity and high grain quality, blast resistance
61	Hyo-kei-sake No. 66 (Toji-no-yume)	2004	Japan	hybridization with mutant variety Hyogo-Kitanishiki obtained by irradiation of seeds	extremly early maturity, high resistance for rice blast disease

				with gamma rays	
62	Shun-you	2004	Japan	hybridization with mutant variety Oochikara induced by irradiation with gamma rays and mutant variety LGC-1 obtained by treatment with chemical mutagen 0.2% EI.	Early-maturity, low proteins, tolerance to lodging, adaptability of direct sowing, moderate field resistance of blast
63	Silky Pearl	2004	Japan	hybridization with mutant variety Yama-uta induced by irradiation of seeds with 200 Gy gamma rays and mutant obtained by treatment with chemical mutagen NMU.	Short culm, low amylose content, superior lodging resistance and good yielding ability, medium leaf and ear-blast resistances
64	Natsushizuka	2005	Japan	hybridization with mutant variety Natsuhikari obtained by irradiation with gamma rays	Extremely early maturity, lodging resistance, non glutinous paddy rice, highly resistant to leaf and panicle blast, and moderately resistant to bacterial leaf blight
65	Sai-no-kagayaki	2005	Japan	hybridization with two mutant varieties Matsuri-bare and Sai-no-yume obtained by irradiation of seeds with gamma rays (200 Gy).	Field resistance to blast, and resistance to stripe disease and green rice leaf hopper, tolerant to lodging with short culm and stiffness, good eating quality
66	Sai-no-kirabiyaka	2005	Japan	hybridization with two mutant varieties Matsuri-bare and Sai-no-yume obtained by irradiation of seeds with gamma rays (200 Gy)	Field resistance to blast, and resistance to stripe disease and green rice leaf hopper, moderately resistance to bacterial leaf blight, tolerant to lodging with extreme short culm and stiffness, good eating quality
67	Tsukushi-roman	2005	Japan	hybridization with mutant variety Yume-tsukushi obtained by irradiation with gamma rays and mutant Chubu No	Early maturity, high and stable palatability, has the blast resistance gene Pii and the field resistances to blast and bacterial leaf blight are weak
68	Churahikari	2006	Japan	hybridization with mutant obtained by mutant variety Mine-asahi irradiated with gamma rays (200 Gy)	High resistance to blast, medium-late maturity, shorter culm and lodging resistance
69	Hana-omoi	2006	Japan	hybridization with mutant variety Hanafubuki obtained by irradiation with gamma rays.	Medium maturity, good quality, suitable for sake brewing
70	Sakihikari	2006	Japan	hybridization with mutant variety Kinuhikari obtained by irradiation with gamma rays	Excellent eating quality, late maturity and show blast resistance

71	Zhongzao 25	2006	China	hybridization with two mutants.	Early maturity, high yield, wide adaptability and blast resistance
72	Huaxiang 7	2007	China	treatment of seeds in aerospace	High yield, good quality and resistance to blast
73	IIyou 623	2007	China	treatment of seeds in aerospace.	Late maturity, medium tillering ability, panicle is large multi-grain, high yield, better grain quality and resistance to blast
74	Dewanosato	2007	Japan	hybridization with mutant variety Dewasansan obtained by irradiation of seeds with gamma rays (300 Gy).	Good quality, suitable for sake brewing and cool weather tolerance
75	Fuku-izumi	2007	Japan	hybridization with mutant variety Dontokoi obtained by irradiation with gamma rays.	Early maturity, short culm, excellent lodging resistance and stable yield
76	Ikuhikari	2007	Japan	hybridization with mutant variety Dontokoi obtained by irradiation with gamma rays	Medium maturity, good eating quality, high yield, show blast resistance, two resistant genes (Pita-2 and Pii), and the field resistance of the leaf and panicle can be rated as medium
77	Yuyakemochi	2009	Japan	hybridization with mutant variety Benigoromo obtained by irradiation of seeds with gamma rays (200 Gy)	Shorter culm, resistance to lodging, resistance to blast grains have higher dietary fiber, sodium, niacin, anthocyanidin, tannin, and catechin content
78	Jianuo1 You No.3	2009	China	hybridization with mutant	High yield, good panicle, resistance to blast, good quality
79	Tsukushi-wase	2015	Malaysia	hybridization with mutant variety Kinuhikari obtained by irradiation with gamma rays	High yield, short culm, resistance to lodging and early maturity, It has the blast resistance gene Pi-i. Field resistances to blast and bacterial leaf blight are week
